# Costs and where to find them: identifying unit costs for health economic evaluations of diabetes in France, Germany and Italy

**DOI:** 10.1007/s10198-020-01229-1

**Published:** 2020-10-06

**Authors:** J. Pöhlmann, K. Norrbacka, K. S. Boye, W. J. Valentine, H. Sapin

**Affiliations:** 1 Ossian Health Economics and Communications, Basel, Switzerland; 2 Eli Lilly Finland, Helsinki, Finland; 3 Eli Lilly and Company, Indianapolis, IN USA; 4 Lilly France, 24 Bd Vital Bouhot, CS 50004, 92521 Neuilly-sur-Seine Cedex, France

**Keywords:** Cost, Cost-effectiveness, Diabetes, France, Germany, Italy, D61, I10

## Abstract

**Background:**

Health economic evaluations require cost data as key inputs. Many countries do not have standardized reference costs so costs used often vary between studies, thereby reducing transparency and transferability. The present review provided a comprehensive overview of cost sources and suggested unit costs for France, Germany and Italy, to support health economic evaluations in these countries, particularly in the field of diabetes.

**Methods:**

A literature review was conducted across multiple databases to identify published unit costs and cost data sources for resource items commonly used in health economic evaluations of antidiabetic therapies. The quality of unit cost reporting was assessed with regard to comprehensiveness of cost reporting and referencing as well as accessibility of cost sources from published cost-effectiveness analyses (CEA) of antidiabetic medications.

**Results:**

An overview of cost sources, including tariff and fee schedules as well as published estimates, was developed for France, Germany and Italy, covering primary and specialist outpatient care, emergency care, hospital treatment, pharmacy costs and lost productivity. Based on these sources, unit cost datasets were suggested for each country. The assessment of unit cost reporting showed that only 60% and 40% of CEAs reported unit costs and referenced them for all pharmacy items, respectively. Less than 20% of CEAs obtained all pharmacy costs from publicly available sources.

**Conclusions:**

This review provides a comprehensive account of available costs and cost sources in France, Germany and Italy to support health economists and increase transparency in health economic evaluations in diabetes.

**Electronic supplementary material:**

The online version of this article (10.1007/s10198-020-01229-1) contains supplementary material, which is available to authorized users.

## Introduction

Costs are key inputs into any health economic evaluation. Depending on the perspective and time horizon of the evaluation as well as available data, costs can be obtained from a variety of sources or calculated using several different approaches [1–5]. As discussed by Hoerger [6] in the context of cost-effectiveness modeling of diabetes, these costing approaches are neither standardized nor straightforward, so the choice of costs may be associated with uncertainty. Similar challenges have been observed, for example, in evaluations of cancer [7], hip fracture [8] and mental illness [9], as well as in health technology assessment (HTA) more broadly [10]. More standardized approaches to costing [11] and even standardized healthcare cost data [12] were suggested to increase transparency and comparability across studies, but articles outlining costing approaches often assume that a researcher has access to multiple sources of costing data, e.g. from institutional databases [11, 13]. Such data may not be available to all researchers and difficult to assess or replicate by reviewers and readers. Instead, publicly available data may be used, which are usually free to access, use and verify, but these data can be difficult to find and are often distributed across multiple sources and platforms.

The present article aims to contribute to the use of publicly available cost sources, by providing an overview of available data sources for unit costs in France [14], Germany [15] and Italy [16], which were chosen as the largest healthcare markets in the European Union in the near future. The study builds on previous cost collection studies [17, 18], and supplements recent efforts, particularly in France and the United Kingdom (UK), to advance costing for healthcare-related studies [1, 2, 13, 19, 20].

In addition to providing an overview of cost data sources, the article presents suggested unit cost sets that can inform health economic analyses in these countries. While most unit costs are anticipated to be applicable to evaluations in different disease areas, the collection of unit costs was structured by requirements for health economic analyses primarily in type 2 diabetes (T2D), which is associated with substantial healthcare costs in all three countries under study. A recent study using French national health insurance data estimated that, in a population of 3 million people with diabetes in 2012, EUR 10 billion in 2012 (of EUR 19 billion in total expenditure) were attributable to diabetes care [21]. Another study, which also used national health insurance data, calculated annual costs of EUR 8.5 billion for patients with T2D in 2013, equivalent to 5% of total health expenditure [22]. For Germany, a cost-of-illness study using statutory health insurance (SHI) data suggested that EUR 16.1 billion, equivalent to approximately 10% of statutory health insurance expenses in Germany, were spent each on the treatment of patients with T2D in 2009–2010 [23]. A cost-of-illness study for Italy estimated direct medical costs to the Servizio Sanitario Nazionale (SSN), the Italian National Health Service, in 2012 at EUR 9.6 billion, with an additional EUR 10.7 billion in indirect costs due to early retirement and absenteeism [24]. Important cost drivers in all three countries were diabetes-related complications, including renal, neuropathic and ophthalmologic complications [25–27], and adverse events, in particular hypoglycemia [28]. Due to the burden associated with T2D in these countries, health economic evaluations in the field of diabetes will continue to play an important role in healthcare resource allocation and decision-making, and the present cost collection contributes data to inform these analyses. As part of this study, the quality of pharmacy unit cost reporting in published cost-effectiveness analysis (CEA) of antidiabetic medications were also reviewed, to identify challenges and evidence gaps related to cost reporting.

## Methods

### Searches to identify unit cost data sources

**Fig. 1:**
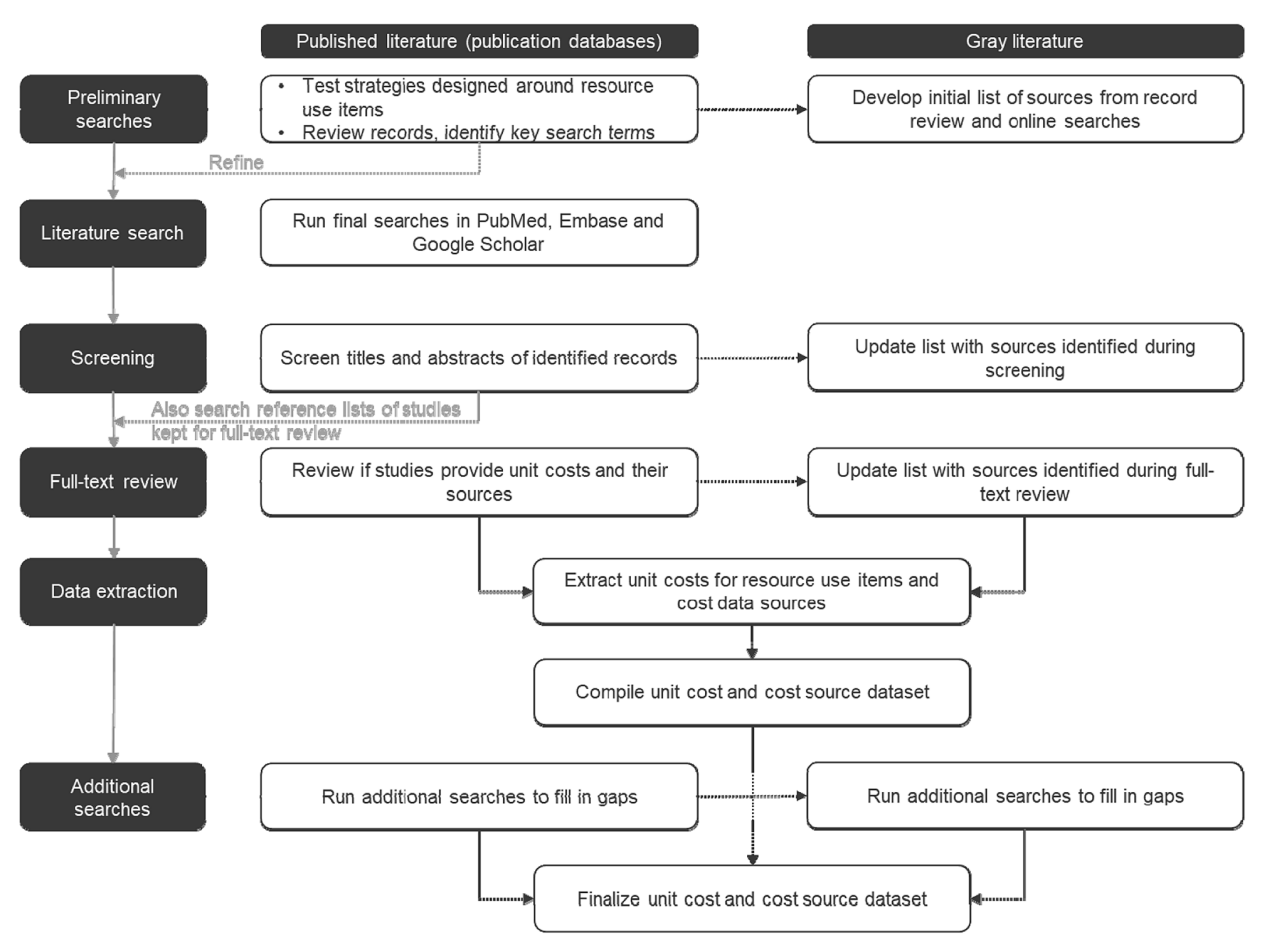
Schematic diagram of the search process to identify unit costs and cost sources

Preliminary searches were developed based on the list of relevant resource items (Table 1) to test their performance and identify studies that could be used to refine the search strings, e.g. by providing additional search terms. Following development of the search strategy in this way, searches were performed in the electronic literature databases PubMed and Embase and in Google Scholar in line with the process detailed in Fig. 1. Final searches were based on the refined preliminary searches and translated into database-specific vocabulary. For PubMed and Embase, searches designed to identify costs for office and diagnosis-related groups (DRGs)/tariffs were conducted without limiting the search to diabetes as many of these costs were not anticipated to vary substantially between patients with and without diabetes (see Online Resources 1–3 for PubMed search strategies). The same broad approach was not feasible for other resource use items as the number of retrieved records would have been beyond the scope of the present project.

**Table 1:** Resource use items of interest

Resource use category	Items of interest
Primary outpatient care	Primary care physician/general practitioner (including practice visit, phone calls)
	Nurse (including practice visit, home visit or phone calls)
	Home or hospital visit (general)
	Diabetes educator or specialized staff
Specialist outpatient care	Cardiologist; dentist; dermatologist; diabetologist/endocrinologist; dietician; nephrologist; neurologist; ophthalmologist; podiatrist; psychiatrist; psychotherapist
Diabetes training or education	Nurse/physician (as applicable)—includes visit at practice, phone calls and home visits
Hospital and inpatient care	Hospital admission (daytime or overnight stay)
	Intensive care unit
Emergency medical care	Emergency department
	Emergency medical services/ambulance transportation
Pharmacy	Medication
	Consumables, including for self-monitoring of blood glucose
Intangible resource use	Lost workplace productivity (caregivers and patients) (daily)

Studies identified in unit cost data searches were eligible for inclusion if they reported unit costs for at least one of the resource items of interest in France, Germany or Italy. Searches targeted costs for populations with diabetes but populations with risk factors for or sequelae of diabetes (e.g. renal disease) were also considered. Studies were not eligible if they were not performed in at least one of the target settings, did not report unit costs or sources of unit costs, were a research protocol or abstract, or were published before 2012 (as costs reported by these studies were considered to be likely obsolete).

Study titles and abstracts were screened according to in- and exclusion criteria by a single researcher. Full-texts of studies retained in the first round of review were then reviewed in depth to determine final eligibility of a study. From eligible studies, unit costs and any information on cost data sources were extracted into a piloted Microsoft Excel workbook to compile a dataset for unit costs and cost sources. Years of reported costs were also extracted to assess how recent costs were at the time of publication and time of search. Additional searches to fill any data gaps were planned but not ultimately required. These data, which were obtained from the literature, were complemented with manual searches of data from HTA, pricing and reimbursement authorities. Searches were last run on 16 October 2018. As only descriptive cost data were extracted from studies, bias assessment was not required.

### Searches to identify health economic evaluations of antidiabetic medication

A separate search was conducted to obtain CEAs to assess the quality of unit cost reporting. Studies were eligible if they reported a CEA (which, for the purposes of the present study, also included cost-utility and cost–benefit analyses) of antidiabetic mediation in patients with diabetes. Studies were considered if they were conducted in any of the three target countries or in Spain or the UK with the latter two countries included to increase the pool of eligible studies while covering the major European healthcare markets. Research protocols and abstracts were excluded as were studies published before 2012 and studies not investigating antidiabetic medications but medical devices (e.g. insulin pumps).

Both PubMed and Embase were searched, with development of search strategies and screening following the procedure outlined above for pharmacy unit cost data (see Online Resource 4 for the PubMed search strategy). Pharmacy costs, specifically the acquisition costs associated with medications, were chosen (as opposed to complication costs, for example) as they could be expected to be included in all analyses, thereby increasing comparability. Three quality indicators were developed and extracted for pharmacy costs from each included CEA, independently by two researchers. First, it was assessed if unit cost values used were reported for all antidiabetic medications under study. Studies were classified as “All”, “In part” or “None” if they reported a unit cost value for all, some or no antidiabetic medication, respectively. Second, it was assessed if used unit costs were referenced so that they could be checked against their source. Studies were again classified as “All”, “In part” or “None” if they provided a clear reference, e.g. a paper or unified resource locator with all the required details to uniquely identify the cost in a database, for all, only some or none of the unit cost values, respectively. Third, it was assessed if cost sources were freely accessible or required a subscription or registration. This criterion was deemed important as transparency relies on accessibility of sources without undue costs or administrative burden. Studies were classified as “All”, “In part” or “None/no references” if all, some or no cost source was freely accessible (with studies providing no cost source references grouped as “None/no references” for this indicator). If a study reported analyses for multiple countries, each country-specific analysis was considered separately.

### Data management and presentation

Cost data were presented in country-specific tables, one each for sources of cost data and for suggested unit costs for the items of interest. The quality assessment of unit cost reporting in CEAs was summarized in descriptive statistics. All data were stored in Microsoft Excel workbooks and, where applicable, analyzed using R version 3.5.1 [29].

## Results

### Overview of search results and study selection

**Fig. 2:**
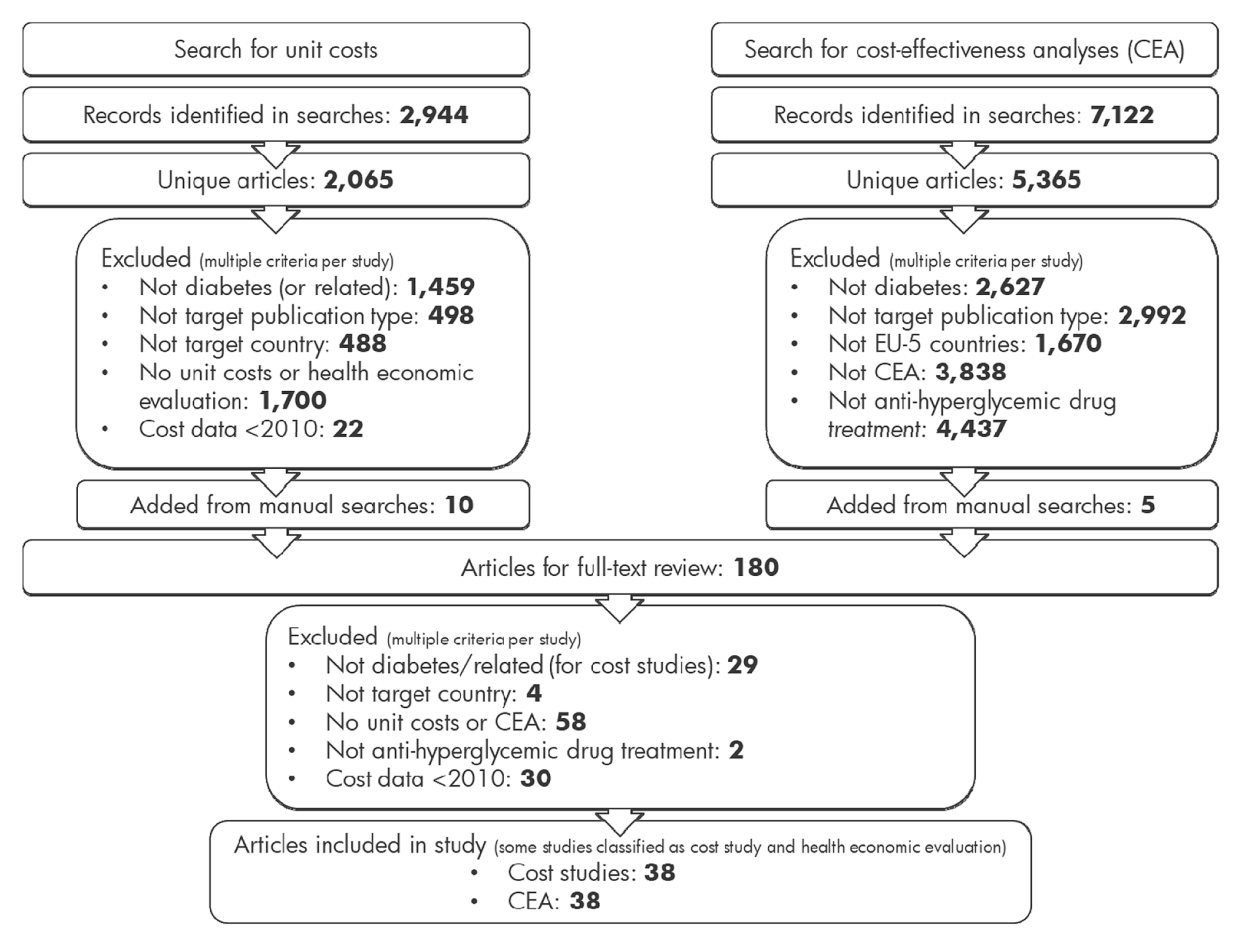
Flowchart for database searches and study selection

The search of literature databases for studies reporting unit costs and cost sources for France, Germany and Italy yielded 2944 hits, of which 2065 were unique articles that were title-and-abstract-screened for full-text review (Fig. 2). All relevant inclusion/exclusion criteria were applied to each article, with the most frequently applied exclusion criteria being that an article did not report unit costs and/or health economic evaluations or was not in diabetes or related diseases. Ten studies were added from manual searches of HTA reports and databases.

With regard to searches for health economic evaluations, 7122 hits, of which 5365 were unique articles, were retrieved from databases (Fig. 2). An additional five studies were obtained from manual searches of HTA reports and databases.

Search results were combined and duplicates removed, yielding 180 articles for full-text review. Criteria for inclusion as a cost-reporting study or health economic evaluation were applied, and each included study was classified as reporting unit costs (or cost sources) and/or a health economic evaluation. In total, 57 studies were included, of which 38 reported unit costs or cost sources and 38 were health economic evaluations.

### Results for unit costs and cost data sources

Of publications reporting unit costs or cost sources, fewer were identified for France (*n* = 9) [30–37] than for Germany (*n* = 15) [23, 38–50] or Italy (*n* = 15) [51–64], with one study reporting data for all three countries [65]. A range of cost sources and unit costs could be obtained from these studies for the resource use items of interest (Table 1).

#### Cost sources for France

France has a heavily centralized healthcare system centered on SHI (Assurance Maladie), which is part of the French Social Security System. The SHI has different schemes that together cover up to 98% of the population [14, 66, 67].

For consultations with primary care and specialist physicians, the SHI publishes “conventional tariffs” for both mainland France and the French overseas territories (Table 2). These tariffs are available for a range of settings (e.g. office versus teleconsultation versus home visit at different times of day/night; regular office hours versus public holidays) and can be used to cost consultations with and visits to physicians. Depending on the type of (procedural) data available to the analyst, e.g., in the context of an observational study, detailed procedural tariffs can be obtained from another SHI source, the Classification Commune des Actes Médicaux (CCAM). The CCAM is based on the French nomenclature for medical acts (Nomenclature Générale des Actes Professionnels, NGAP) and contains consultations and clinical procedures, specified by keywords, procedure code or clinical field and based on regularly updated nomenclature, in addition to monetary values for each procedure.

**Table 2:** Overview of cost sources for France

Item	Source				Comment
	Name	Publisher	Description	Access and update	
*Physicians: consultations, visits, outpatient procedures*					
Consultations (including home visits) for GPs and specialists	Tarifs conventionnels	Ameli	Standard tariffs for consultations Available for GPs and specialists, for consultations in the office and for home visits	Free, online at ameli.fr/medecin/exercice-liberal/facturation-remuneration/tarifs Updated regularly	
Clinical outpatient procedures	CCAM, based on NGAP	Ameli	Coding and SHI tariffs of most medical procedures	Free; online at ameli.fr/accueil-de-la-ccam/index.php Updated regularly	
*Ambulance transport and emergency treatment*					
EMS	SMUR tariffs	Various hospitals, particularly CHUs	Tariffs for land and air EMS, per half-hour (for land transport) and per minute (for air transport)	Free, online, e.g. at CHU Bordeaux (chu-bordeaux.fr/CHU-de-Bordeaux/Publications-l%C3%A9gales/Guide-de-la-tarification/Guide-de-la-tarification-2018.pdf/) Update cycles vary depending on respective hospital/CHU	Available for different CHUs Base d’Angers (CHU Angers) provides hospital perspective on (actual) SMU costs RTC with reference cost for SMUR (codes Q021 and Q023)
Planned patient transport	Transporteur sanitaire: tariffs	Ameli	Standard tariffs for planned patient transports, with varying levels of patient support needed	Free, online at ameli.fr/transporteur-sanitaire/exercice-professionnel/facturation/tarifs Updated regularly but infrequently	
Emergency department	RTC	ATIH	Repository for hospital-related unit costs, providing costs per case averaged over participating institutions	Free, online at scansante.fr/applications/cout-dunites-doeuvre (item 932112—Accueil et traitements des urgences de Médecine) Updated regularly but infrequently	
*Hospitalization*					
Hospital, including ICU	Tarifs MCO, HAD	ATIH	GHS with coding based on the CCAM	Free, online at atih.sante.fr/tarifs-mco-et-had Updated regularly, usually yearly	RTC with reference costs by clinical field such as diabetology (code 93,413,162) or intensive care (code 9,341,429)
*Medication (drugs and consumables)*					
Drugs	Thesorimed®	GIE-SIPS	Factory and public price data (including tax), reimbursement rate and generic versions	Free, online at theso.prod-un.thesorimed.org/monographie Updated regularly	Also available from Base des Médicaments et Informations Tarifaires
Consumables	Liste des Produits et des Prestations	Ameli	Medical device tariffs	Free, online at codage.ext.cnamts.fr/codif/tips/index_presentation.php?p_site = AMELI Updated regularly	
*Intangible costs*					
Wages	Average wage	OECD	Annual average wage and hours worked data	Free, online at https://data.oecd.org/earnwage/average-wages.htm and https://data.oecd.org/emp/hours-worked.htm Updated regularly	Data provided in US dollars for all countries, which increases comparability

Ameli, Assurance Maladie; ATIH, Agence Technique de l’Information sur l’Hospitalisation; CCAM, Classification Commune des Actes Médicaux; CHU, Centre Hospitalier Universitaire; EMS, Emergency Medical Services; GIE-SIPS, Groupement d'Intérêt Economique—Système d'Information sur les Produits de Santé; GHS, Groupes Homogènes de Séjours; GP, General Practitioner; HAD, Hospitalisation à Domicile; ICU, Intensive Care Unit; INSEE, Institut National de la Statistique et des Études Économiques; MCO, Médecine, Chirurgie, Obstétrique; NGAP, Nomenclature Générale des Actes Professionnels; OECD, Organization for Economic Co-operation and Development; RTC, Référentiel de Coût des Unités d'Oeuvres; SHI, Statutory Healthcare Insurance; SMUR, Structure Mobile d'Urgence et de Réanimation

Responsibility for emergency medical services (EMS), in the form of either land or air rescue (Structure Mobile d’Urgence et de Reanimation, SMUR), rests with hospitals as part of regional emergency care infrastructures. SMUR services are frequently provided by university hospitals (Centre Hospitalier Universitaire, CHU) and SMUR tariffs can be obtained from the websites of various CHUs. For planned patient transports, with varying levels of patient supported required, SHI tariffs are available. Of note, the cost database Base d’Angers provides actual SMUR cost data from its participating institutions in France. This database could be used to complement the tariffs outlined in the sources above.

Costs associated with emergency department (ED) treatment can be sourced from national unit cost reference data (Référentiel de Coût des Unités d'Oeuvres, RTC). The RTC provides unit costs calculated from participating institutions for a wide range of clinical, technical and logistical services related to healthcare, including costs associated with emergency medical treatment (e.g. code 932112—Admission and treatment of medical emergencies). The data are available online and freely accessible, with the latest data from 2016. For hospitalization, including stays in intensive care units (ICU), tariffs for the French DRGs, the Groupes Homogènes de Séjours, are freely available online from the Technical Agency for Information on Hospital Stays (Agence Technique de l’Information sur l’Hospitalisation). Again, actual costs from participating institutions are also available from the RTC.

Pharmacy and consumable costs in France are also freely available online. These costs can be accessed using a variety of different interfaces. For pharmacy costs, Thesorimed^®^ provides a modern user interface and information on a medication’s reimbursement status, costs (including and excluding taxes), generics and equivalents, and reimbursement decisions. For medical devices, costs can be accessed from the list of products and benefits (Liste des Produits et des Prestations).

Regarding intangible resource use, measured as time lost and valued using wages, this can be obtained from the Organization for Economic Co-operation and Development (OECD), which may be preferred if wage data are required for multiple countries to ensure consistency and comparability of calculations. Similar data are provided in the official wage and labor statistics published by the National Institute of Statistics and Economic Studies (Institut National de la Statistique et des Études, INSEE). The INSEE provides longitudinal gross and net wage data, which are freely accessible online.

Based on the cost sources identified for France, a suggested unit cost dataset (Online Resource 6) was developed for the resource items of interest. This dataset, which mirrors and updates previous work for France [36], can be used in its current form or as a starting point for a dedicated cost collection.

#### Cost sources for Germany

The German healthcare system is centered on statutory and private healthcare insurance (PHI) [15]. Statutory insurance is corporatist and mostly self-regulated on behalf of the government in negotiations by sickness funds (payers) and physician, dentist and hospital associations (providers). A distinctive feature of the German healthcare system is the existence of full-cover PHIs. While PHIs use the same DRG schedule as statutory sickness funds, they differ in fee schedules for physicians. Cost data for both SHI and PHI are presented although the focus is on SHI, which covers approximately 80% of the population [15].

Cost data for consultations with primary care or specialist physicians (in the office, at home or via phone) are available from the Uniform Value Scale (Einheitlicher Bewertungsmassstab, EBM) from an SHI perspective (Table 3). The EBM is a database of procedures and services that physicians may charge SHI. The data are available online and freely accessible. Equivalent data for the PHI perspective can be sourced from fee schedules for physicians (Gebührenordnung für Ärzte, GOÄ) and dentists (Gebührenordnung für Zahnärzte, GOZ), respectively. Both are freely available online. In addition to these databases, a frequently referenced source of unit costs, including for visits to physicians, is the study by Bock et al*.* [39]. In this study, the authors calculated “valuation rates” for physician–patient contacts based on overall budgets paid to physicians by sickness funds and contact data, while also including PHI data. These valuation rates are used frequently in German health economic evaluations covering a range of disease areas due to their convenience and granularity [48, 68, 69]. Similar data, providing average remuneration per case, are provided by the National Association of Statutory Health Insurance Physicians (Kassenärztliche Bundesvereiningung) in their yearly report. For consultations and services provided by non-medical personnel such as podologists, tariff lists are available online from sickness fund associations (e.g. Verband der Ersatzkassen).

**Table 3:** Overview of cost sources for Germany

Item	Source				
	Name	Publisher	Description	Access and update	Comment
*Physicians: consultations, visits, outpatient procedures*					
Consultations (including home visits) and outpatient procedures for GPs and specialists	Einheitlicher Bewertungsmassstab	SHI (represented by KBV)	Medical and related procedures in outpatient setting, covered by SHI	Free; online at kbv.de/html/online-ebm.php Updated regularly	
Consultations (including home visits) and outpatient procedures for GPs and specialists	Standardisierte Bewertungssätze	SHI (represented by KBV)	Standardized costs, based on SHI expenditure, per physician–patient contact	Bock et al*.* 2015, Gesundheitswesen Update possible based on methods outlined in the article	Used frequently by publications in German setting
Consultations (including home visits) and outpatient procedures for GPs, specialists and dentists	Gebührenordnung für Zahnärzte, Gebührenordnung für Zahnärzte	PHI	Medical and related procedures in outpatient setting, covered by PHI	Free; online at e-bis.de/goae/defaultFrame.htm and bzaek.de/fileadmin/PDFs/goz/gebuehrenordnung_fuer_zahnaerzte_2012.pdf Updated regularly	
Consultations and outpatient procedures for non-physicians	Preisvereinbarungen und Vergütungslisten bei Heilmitteln	SHI (represented by vdek)	Tariffs for medical procedures performed by non-physicians	Free; online at vdek.com/vertragspartner/heilmittel/preisvereinbarungen.html Updated regularly	
*Ambulance transport*					
EMS and planned patient transport	Gebührensatzung Rettungsdienst	Kreis Euskirchen (Northrine-Westphalia)	Fee schedule for EMS and planned patient transport	Free, online at https://www.kreis-euskirchen.de/service/downloads/gefahrenvorsorge/5_322GebuehrSatzgRD.pdf Updated regularly but frequency dependent on county/region	No standardized EMS fee structure in Germany Similar data available for other counties/regions
*Hospitalization (including ER and ICU treatment)*					
Hospital	G-DRG-Fall-pauschalen-Katalog	SHI (represented by InEK)	DRGs for inpatient procedures, including their coding and costs	Free, online at https://www.g-drg.de/G-DRG-System_2018/Fallpauschalen-Katalog/Fallpauschalen-Katalog_2018 Updated regularly, usually yearly	Monetary value of base rates provided by vdek (vdek.com/vertragspartner/Krankenhaeuser/landesbasisfallwerte.html)
*Medication (drugs and consumables)*					
Drugs and consumables	Lauer-Taxe® Rote Liste®	Lauer-Fischer GmbH Rote Liste® Service GmbH	Databases for drug and consumable prices	Proprietary, online at cgm.com/lauer-fischer/loesungen_lf/lauer_taxe_lf/lauer_taxe.de.jsp and rote-liste.de/ Updated regularly	Databases require paid subscription
*Intangible costs*					
Wages	Average wage	OECD	Annual average wage and hours worked data	Free, online at https://data.oecd.org/earnwage/average-wages.htm and https://data.oecd.org/emp/hours-worked.htm Updated regularly	Data provided in US dollars for all countries, which increases comparability

DRG, Diagnosis-Related Group; EMS, Emergency Medical Services; GP, General Practitioner; KBV, Kassenärztliche Bundesvereinigung; OECD, PHI, Private Healthcare Insurance; SHI, Statutory Healthcare Insurance; vdek, Verband der Ersatzkassen

Costs of ambulance transportation are regulated at the local and regional level, so no nationwide applicable fees exist. Examples identified during the review include recent fee schedules for Euskirchen county (in North Rhine-Westphalia), but schedules covering other municipalities and regions are also available. Emergency department treatment in Germany is performed in hospitals and, therefore, covered by the G-DRG system, as are inpatient stays and ICU treatment. Both the G-DRG system and a list of monetary base rate values are freely accessible online.

Unlike in France and Italy, pharmacy cost data are not freely accessible in Germany. Instead, a paid subscription is required to access databases such as the Lauer-Taxe^®^ or the Rote Liste^®^. Drug prices from 1 year after market introduction can be approximated using reimbursement prices from price-setting negotiations. However, these prices do not reflect prices during the first year after market introduction, sickness fund-specific rebates and other changes to prices. Data on wages and hours worked can be sourced from the OECD but are also available from the Federal Statistical Office (Destatis).

Based on the cost sources identified for Germany, a suggested unit cost dataset was developed for the resource items of interest (Online Resource 7).

#### Cost sources for Italy

The Italian healthcare system is highly decentralized. Within the SSN, implementation and delivery of healthcare rests with the 21 regions and provinces. Most costs are, therefore, available from different regions. In the cost dataset compiled for Italy, costs were presented for the Bolzano, Emilia-Romagna, Umbria and Apulia provinces/regions to obtain a broad geographic spread across the country.

Costs of consultations, including primary and specialist care in the office and for home visits, can be sourced from regional tariffs (Nomenclatore tariffario regionale) (Table 4). Tariff lists are generally freely available online and updated regularly although update frequency differs between regions.

**Table 4:** Overview of cost sources for Italy

Item	Source				
	Name	Publisher	Description	Access and update	Comment
*Physicians: consultations, visits, outpatient procedures*					
Consultations (including home visits) and outpatient procedures for GPs and specialists	Nomenclatore tariffario regionale	Regional and provincial health authorities	Coding of and tariffs for outpatient procedures performed by physicians	Free, online at respective websites, e.g. for Bolzano (provinz.bz.it/gesundheit-leben/gesundheit/downloads/1_Prestazioni_di_specialistica_ambulatoriale_ex_DM_22.07.1996.pdf) or Emilia-Romagna (salute.regione.emilia-romagna.it/documentazione/leggi/regionali/dgr-2127–2016/delibere-specialistica) Updated regularly but frequency dependent on respective region/province	
*Ambulance transport and emergency treatment*					
EMS	Sanita24	Sanita24	Cost of land rescue	Free, online at sanita24.ilsole24ore.com/art/regioni-e-aziende/2015–02-12/emergenza-fiaso-ecco-costi-113448.php?uuid = AbcvOTBL Last updated in 2015	Used in recent literature, e.g. Giorda et al*.* 2017, Nutr Metab Cardiovasc Dis
Ambulance transportation	Tariffario per trasporti socio-sanitari non urgenti	Croce Rossa Italiana (Italian Red Cross)	Tariffs (flat rates) for transports of different distances	Free, online at cri-itri.it/wp-content/uploads/2016/09/Clicca-qui-per-scaricare-le-nostre-tariffe-2016.pdf Last updated in 2016	
Emergency department	Progetto Mattoni SSN: pronto soccorso e Sistema 118	SSN	Assessment of emergency department costs	Free, online at mattoni.salute.gov.it/mattoni/documenti/11_Valutazione_costi_dell_emergenza.pdf Last updated in 2007	Used in recent literature, e.g. Giorda et al*.* 2017, Nutr Metab Cardiovasc Dis
*Hospitalization*					
Hospital, including ICU	Tariffe DRG	Regional and provincial health authorities	Coding of and tariffs for inpatient procedures	Free, online at respective websites, e.g. for Bolzano (provinz.bz.it/gesundheitswesen/download/delibera_228__13_02_2012.pdf) or Emilia-Romagna (salute.regione.emilia-romagna.it/siseps/sanita/sdo/files/allegato_3_TARIFFE_DRG.xls/view) Updated regularly but frequency dependent on respective region/province	
*Medication (drugs and consumables)*					
Drugs	Liste di trasparenza e rimborsabilità	AIFA	List of drugs (with prices) reimbursed by SSN	Free, online at www.agenziafarmaco.gov.it/content/liste-di-trasparenza-e-rimborsabilit%C3%A0 Update regularly	Association of SSN-affiliated pharmacies (Federfarma) provides public prices (federfarma.it/Farmaci-e-farmacie/Cerca-un-farmaco.aspx)
Consumables	Costi unitari dispositivi medici	Regional and provincial health authorities	List of unit costs for consumables	Free, online at respective websites, e.g. for ASST Garda (asst-garda.it/amministrazione-trasparente/costi-unitari-dei-dispositivi-medici/) or for ASST Bergamo Est (bolognini.bg.it/ITA/Default.aspx?SEZ = 1&PAG = 100) Updated regularly but frequency dependent on respective region/province	List of medical devices available provided by the Ministry of Health (www.dati.salute.gov.it/dati/dettaglioDataset.jsp?menu=dati&idPag=1)
*Intangible costs*					
Wages	Average wage	OECD	Annual average wage and hours worked data	Free, online at https://data.oecd.org/earnwage/average-wages.htm and https://data.oecd.org/emp/hours-worked.htm Updated regularly	Data provided in US dollars for all countries, which increases comparability

AIFA, Agenzia Italiana del Farmaco; ASST, Azienda Socio Sanitaria Territorial; DRG, Diagnosis-Related Group; EMS, Emergency Medical Services; GP, General Practitioner; ICU, Intensive Care Unit; Istat, Istituto Nazionale di Statistica; OECD, Organization for Economic Co-operation and Development; SSN, Servizio Sanitario Nazionale

By comparison, cost data for EMS and planned patient transports as well as ED treatment costs are more difficult to obtain. For EMS, a commonly used reference in the literature for Italy is based on a costing study covering the Basilicata, Emilia-Romagna, Lazio and Lombardy regions, which is used in the recent literature [58]. For planned patient transports, the Italian Red Cross provides flat-rate tariffs per transport that differ by vehicle type, staffing and number of journey and are supplemented with mileage allowances. For ED treatment, unit costs published in 2007 by the Progetto Mattoni SSN are still in use despite their age [58].

Regional DRG tariffs are available for inpatient treatment, including ICU treatment. These are freely accessible online, with update frequency and convenience of access differing between regions. Current pharmacy costs are also freely available online, from the Italian Medicines Agency (Agenzia Italiana del Farmaco), which provides an SSN perspective, while the Italian Federation of Pharmacies provides a database of public prices. Consumables covered by SSN were listed by the Ministry of Health, while corresponding cost data were again provided by regional and local health authorities. Data on wages and hours worked, based on national labor agreements, are available from the National Statistics Institute (IStat) and the OECD.

Based on the cost sources identified for Italy, a suggested unit cost dataset was developed for the resource items of interest (Online Resource 8).

#### Quality of pharmacy cost reporting in CEAs

**Fig. 3:**
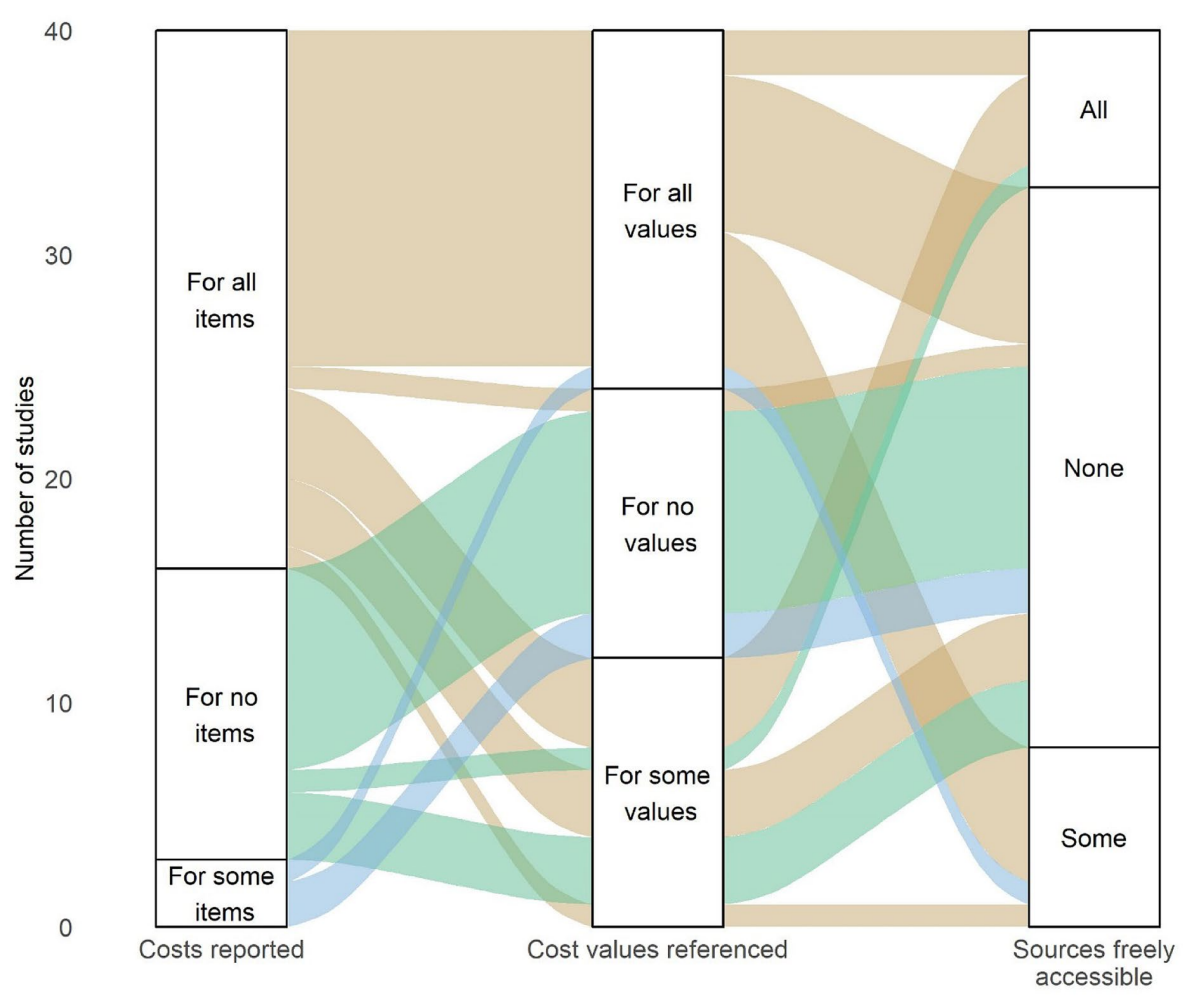
Quality of pharmacy unit cost reporting. Note: costs reported and referenced refers to reporting of pharmacy unit costs

Overall, 38 studies reporting health economic analysis of antidiabetic medications were included, with one study reporting evaluations for three countries of interest [30, 51, 70–105]. Of the 40 evaluations, most were for the UK (*n* = 23), followed by Spain (*n* = 9) and Italy (*n* = 4), with two each from France and Germany. With regard to reporting pharmacy unit costs used, 60% (*n* = 24) of evaluations reported all, while 7.5% (*n* = 3) reported only some and 32.5% (*n* = 13) reported none of the unit costs for included antidiabetic medications. All costs were referenced clearly by 40% (*n* = 16), whereas 30% (*n* = 12) provided references for only some or none of the costs. In 17.5% (*n* = 7) of evaluations, all pharmacy costs were obtained from freely accessible sources while some non-freely accessible sources were used in 20% (*n* = 8) of evaluations, with the remainder either not referencing any pharmacy cost or using costs that were not freely accessible. Overall, only two evaluations (5%) reported and referenced all pharmacy costs and used freely accessible sources, while nine (22.5%) did neither report nor reference any pharmacy unit costs (Fig. 3).

## Discussion

The present review was designed to provide a comprehensive, practical overview of cost data sources and unit costs suitable for health economic evaluations in the field of diabetes, for France, Germany and Italy. Both sources and costs were obtained from published studies and the gray literature, and included tariff and fee schedules for physician consultations (in the office and at home), out- and inpatient procedures, EMS and ED treatment as well as pharmacy prices and valuations of lost productivity based on wages.

Some differences between countries regarding availability, ease-of-access and comprehensiveness of cost sources were observed. In a centralized healthcare system such as in France, SHI and nationwide data sources were available for almost all resource items of interest. While these sources were generally found to be current and updated regularly (with many available in modern user interfaces), they were complex to use, often requiring an intimate knowledge of the French healthcare system [13, 19, 66]. In addition, recent transitions between classification systems and the number of different data sources available further increased the complexity of obtaining data and targeting the most relevant information. Of note, several large-scale clinical and cost databases were available in France, in particular the nationwide Système National d’Information Interrégimes de l’Assurance Maladie (SNIIRAM) which will be expanded to national health data system over the next years [66, 67]. The demographic, health and cost data provided by SNIIRAM and its subset, the Echantillon Généraliste de Bénéficiaires (EGB), were frequently used in the healthcare literature for France [21, 35, 36]. These sources were considered to be of high quality and to likely represent the best data choice for real-world studies of costs, but were not necessarily suitable for health economic evaluations, particularly those involving modelling. Use of the SNIIRAM and EGB databases is complex and requires prior approval by the steering committee as data are not publicly available, thereby limiting transparency. Published SNIIRAM cost estimates, in turn, were usually population specific and not suitable for use as unit costs although aggregate SNIIRAM costs may inform, for example, the costing of diabetes-related complications (which is beyond the scope of the present study).

For Germany, which has a corporatist healthcare system, nationwide tariff and fee schedules were also identified, many of them available online and in modern user interfaces. A distinctive feature of the healthcare system in Germany was the important role played by PHI, so data could be sourced from different sources depending on the perspective of interest. An example was the costing of physician consultations and outpatient procedures, which would be performed using the EBM from the SHI perspective and the GOÄ/GOZ from the PHI perspective. Unlike France and Italy, however, drug prices were not freely available for the German setting as the two most popular databases required paid subscriptions. Prices could be approximated using publicly available data from price setting negotiations, but these would not account for rebates and not necessarily reflect current prices.

In contrast, multiple sources for each resource use item were available in the decentralized Italian healthcare system. While item coding was usually consistent across healthcare regions and unit costs were often similar, researchers would still be required to decide on the healthcare region to which obtain data from. As became evident during the review, regions differ in the quality and usability of their healthcare cost data available online, which may influence the choice of data. With the ARNO Observatory and Associazione Medici Diabetologi Annals, large-scale, diabetes-specific databases of clinical outcomes and costs for patients were also available [52, 61, 106]. Similar to the SNIIRAM and EGB databases, however, their use would be very complex and require prior approval while published aggregate costs from these sources would not usually be suitable as unit costs.

Common to all three countries were differences in data availability and quality between items. Tariff and fee schedules covering activities of physicians and inpatient procedures were straightforward to identify and updated regularly. In contrast, data covering non-physician medical staff, e.g. nurses, or EMS were harder to obtain and generally of poorer quality. Particularly in the case of EMS, this likely reflected the absence of central planning or reimbursement, for which responsibility often laid with private or non-governmental organizations.

Despite its comprehensiveness, the present review was not without limitations. The review did not consider different costing approaches, many of which have been discussed in the literature, particularly for France [1–4, 11]. Such a methodologic discussion may be relevant for some studies, including those where bottom-up costing approaches may be feasible. In contrast, the present study was focused on providing a practical overview of different cost sources, especially targeting health economics research where costs need to be obtained from an external source, e.g. in case of long-term modeling. A frequent challenge in this context is the use of tariffs and fee schedules such as the German EBM or DRG in the absence of detailed procedural data for a patient population. In this case, assumptions have to be made regarding the likely procedures that an average patient may have undergone, e.g. during a consultation with a diabetologist, as was done in the present study for developing suggested unit cost datasets (Online Resources 6−8). While assumptions introduce uncertainty, particularly with regard to overall costs or budget impact, their impact might be smaller for comparative outcomes such as cost-effectiveness as incremental differences would not be affected as long as the same cost set was used in all arms of the analysis. An additional limitation was the restriction of literature searches to studies reporting costs or cost sources in the context of diabetes. This restriction, which was applied to keep searches practicable, may have implied that potential costs or cost sources were missed. However, as searches covered several years of published studies in both English and the local language and were supplemented with searches of reference lists and the gray literature, the risk of missing costs or sources was considered small.

The overview of cost sources and suggested unit cost datasets was designed not only to provide researchers with a starting point for their analyses and cost collections but also to increase the transparency and accessibility of costs in health economic evaluations [6, 11, 20]. As part of the present study, the quality of unit cost reporting of pharmacy costs in CEAs of antidiabetic medications in the five largest European healthcare markets was assessed. Few studies were found to report and reference costs in full while using cost sources that were freely accessible. These findings did not imply that authors deliberately tried to obfuscate data as, instead, journal restrictions on the number of tables or word limits are much more likely to be responsible for the lack of comprehensive reporting. However, the use of non-standardized costs and difficulties in assessing their origin limit the transparency and transferability of health economic evaluations, particularly in the context of HTA [107, 108]. The value of a more standardized approach to HTA methods was discussed previously [109] and likely extends to HTA input data, including costs [110]. An overview of available unit costs and cost sources, in addition to their transparent reporting, can provide a first step towards more transparent HTA and health economic evaluations.

## Conclusion

This review provided cost data sources and unit costs for use in health economic evaluations in France, Germany and Italy. Differences between countries were observed in ease-of-access for and complexity of cost databases, which partly reflected the structure of the respective health care system. Similarly, differences were observed between resource use items, with more cost data available for in- and outpatient procedures than for EMS or activities performed by non-physician medical staff. The resources highlighted in this study could be used to support health economists in obtaining country-specific cost data required for modeling, particularly in the field of diabetes. Cost collection studies of this type can contribute to increased transparency and standardization of cost data used in health economics and HTA.

## Electronic supplementary material

Below is the link to the electronic supplementary material.

Supplementary file 1 (DOCX 58 kb)
