# Supplementary material for: Costs and where to find them: identifying unit costs for health economic evaluations of diabetes in France, Germany and Italy
Source: Eur J Health Econ. 2020 Oct 6;21(8):1179–96. doi: 10.1007/s10198-020-01229-1 (PMC7561572; doi:10.1007/s10198-020-01229-1)
Supplement: Supplementary file 1 — (DOCX 58 kb) [file 10198_2020_1229_MOESM1_ESM.docx]

Electronic supplementary material

**Online Resource 1: Main unit cost search strategy for PubMed**

|  | **Description** | **Search term** |
| --- | --- | --- |
| #1 | Population with diabetes | diabetes mellitus OR (type 1 diabetes OR diabetes type 1 OR insulin-dependent diabetes) OR (type 2 diabetes OR diabetes type 2 OR non-insulin dependent diabetes) |
| #2 | Cost (of illness) and health economic studies | "Costs and Cost Analysis"[Mesh] OR "costing system" OR economics OR cost OR fee schedule OR expenditure OR "Health Resources"[Mesh] OR "Equipment and Supplies/economics"[MeSH] OR "Equipment and Supplies/manpower"[MeSH] OR "Equipment and Supplies/nursing"[MeSH] OR "Equipment and Supplies/statistics and numerical data"[MeSH] OR "Equipment and Supplies/utilization"[MeSH] OR "indirect cost" OR "unit cost" OR "unit price" OR "price" OR "cost" OR "charge" OR "payment" OR "tariff" OR "budget" OR "budget impact" OR "spending" OR economic model |
| #3 | Primary care | therapy OR treatment OR "Primary Health Care"[Mesh] OR primary care OR referral OR consultation OR house calls OR office visit OR "office visit" OR "practice visit" OR "physician visit" OR "Specialties, Nursing"[Mesh] OR "Home Care Services"[Mesh] OR outpatient OR telemedicine OR "Health Personnel"[Mesh] OR physician OR general practitioner |
| #4 | Specialist outpatient care | diabetologist OR endocrinologist OR dietician OR ophthalmologist OR podiatrist OR cardiologist OR nephrologist OR neurologist OR psychiatrist OR psychotherapist OR dermatologist OR dentist |
| #5 | Training/ education | "Patient Education as Topic"[Majr] OR "patient education"[All Fields] OR "diabetes educator"[All Fields] OR "Health Knowledge, Attitudes, Practice"[Mesh] |
| #6 | Hospital and inpatient care | hospitalisation OR length of stay OR inpatients OR intensive care unit OR emergency service OR transportation of patients[Mesh] |
| #7 | Pharmacy | "pharmaceutical preparations"[Mesh] OR ("pharmaceutical" AND "preparations") OR "pharmaceutical preparations" OR "medication" or "drug" OR "drug therapy/economics"[MeSH] OR "drug therapy/statistics and numerical data"[MeSH] OR "drug therapy/utilization"[MeSH] OR Blood Glucose Self-Monitoring OR "SMBG" OR "continuous glucose monitoring" OR "CGM" |
| #8 | Intangible resources | employment OR absenteeism OR presenteeism OR sick leave OR medical leave OR "loss of productivity" OR "productivity loss" |
| #9 | Limit to France, Germany, Italy | (France[MeSH] OR France[tw] OR French[tw] OR Francais[tw] OR Paris[tw] OR Marseille[tw] OR Lyon[tw] OR Toulouse[tw] OR Nice[tw] OR Nantes[tw] OR Strasbourg[tw] OR Montpellier[tw] OR Bordeaux[tw] OR Lille[tw] OR Germany[MeSH] OR Germany[tw] OR German[tw] OR "Federal Republic of Germany"[tw] OR "German Federal Republic"[tw] OR Deutschland[tw] OR Deutsch[tw] OR Berlin[tw] OR Hamburg[tw] OR Munich[tw] OR München[tw] OR Cologne[tw] OR Köln[tw] OR Frankfurt[tw] OR Stuttgart[tw] OR Düsseldorf[tw] OR Dortmund[tw] OR Essen[tw] OR Leipzig[tw] OR Italy[MeSH] OR Italy[tw] OR Italian[tw] OR Italia[tw] OR Rome[tw] OR Roma[tw] OR Milan[tw] OR Milano[tw] OR Naples[tw] OR Napoli[tw] OR Turin[tw] OR Torino[tw] OR Palermo[tw] OR Genoa[tw] OR Genova[tw] OR Bologna[tw] OR Florence[tw] OR Firenze[tw] OR Bari[tw] OR Catania[tw]) |
| #10 | Combine cost-related terms (outcomes) | #2 AND #3 AND #4 AND #5 AND #6 AND #7 AND #8 |
| #11 | Combine with population terms | #10 AND #1 |
| #12 | Combine with country terms | #11 AND #9 |
| #13 | Published since 2012 | #12 AND ("2012/01/01"[PDat] : "2018/12/31"[PDat]) |

**Online Resource 2: Office cost search for PubMed**

|  | **Description** | **Search term** |
| --- | --- | --- |
| #1 | Setting and activity | (office OR specialist OR physician OR general practitioner OR diabetologist OR endocrinologist OR dietician OR ophthalmologist OR podiatrist OR cardiologist OR nephrologist OR neurologist OR psychiatrist OR psychotherapist OR dermatologist OR dentist) AND (visit OR consultation OR referral OR call) |
| #2 | Cost | cost OR economic OR fee OR payment |
| #3 | Limit to France, Germany, Italy | (France[MeSH] OR France[tw] OR French[tw] OR Francais[tw] OR Paris[tw] OR Marseille[tw] OR Lyon[tw] OR Toulouse[tw] OR Nice[tw] OR Nantes[tw] OR Strasbourg[tw] OR Montpellier[tw] OR Bordeaux[tw] OR Lille[tw] OR Germany[MeSH] OR Germany[tw] OR German[tw] OR Federal Republic of Germany[tw] OR German Federal Republic[tw] OR Deutschland[tw] OR Deutsch[tw] OR Berlin[tw] OR Hamburg[tw] OR Munich[tw] OR München[tw] OR Cologne[tw] OR Köln[tw] OR Frankfurt[tw] OR Stuttgart[tw] OR Düsseldorf[tw] OR Dortmund[tw] OR Essen[tw] OR Leipzig[tw] OR Italy[MeSH] OR Italy[tw] OR Italian[tw] OR Italia[tw] OR Rome[tw] OR Roma[tw] OR Milan[tw] OR Milano[tw] OR Naples[tw] OR Napoli[tw] OR Turin[tw] OR Torino[tw] OR Palermo[tw] OR Genoa[tw] OR Genova[tw] OR Bologna[tw] OR Florence[tw] OR Firenze[tw] OR Bari[tw] OR Catania[tw]) |
| #4 | Combine setting-plus-activity and cost terms | #1 AND #2 |
| #5 | Combine with country terms | #4 AND #3 |
| #6 | Published since 2012 | #5 AND (2012/01/01[PDat] : 2018/12/31[PDat]) |

**Online Resource 3: Diagnosis-related group and tariff search for PubMed**

|  | **Description** | **Search term** |
| --- | --- | --- |
| #1 | DRG and tariffs | (diagnosis related group OR tariff) AND health care |
| #2 | Limit to France, Germany, Italy | France[MeSH] OR France[tw] OR French[tw] OR Francais[tw] OR Paris[tw] OR Marseille[tw] OR Lyon[tw] OR Toulouse[tw] OR Nice[tw] OR Nantes[tw] OR Strasbourg[tw] OR Montpellier[tw] OR Bordeaux[tw] OR Lille[tw] OR Germany[MeSH] OR Germany[tw] OR German[tw] OR Federal Republic of Germany[tw] OR German Federal Republic[tw] OR Deutschland[tw] OR Deutsch[tw] OR Berlin[tw] OR Hamburg[tw] OR Munich[tw] OR München[tw] OR Cologne[tw] OR Köln[tw] OR Frankfurt[tw] OR Stuttgart[tw] OR Düsseldorf[tw] OR Dortmund[tw] OR Essen[tw] OR Leipzig[tw] OR Italy[MeSH] OR Italy[tw] OR Italian[tw] OR Italia[tw] OR Rome[tw] OR Roma[tw] OR Milan[tw] OR Milano[tw] OR Naples[tw] OR Napoli[tw] OR Turin[tw] OR Torino[tw] OR Palermo[tw] OR Genoa[tw] OR Genova[tw] OR Bologna[tw] OR Florence[tw] OR Firenze[tw] OR Bari[tw] OR Catania[tw] |
| #3 | Published since 2012 | #1 AND #2 AND (2012/01/01[PDat] : 2018/12/31[PDat]) |

**Online Resource 4: Cost-effectiveness analyses search for PubMed**

|  | **Description** | **Search term** |
| --- | --- | --- |
| #1 | Diabetes | diabetes[tw] |
| #2 | Health economic evaluations | (cost-effectiveness[tw] OR cost-utility[tw] OR cost-benefit[tw] OR "Cost-Benefit Analysis"[Mesh]) |
| #3 | Published since 2012 | #1 AND #2 AND (2012/01/01[PDat] : 2018/12/31[PDat]) |

**Online Resource 5: Characteristics of studies reporting unit costs and/or cost sources**

| Study | Study aim (population) | Cost perspective and year | Relevant items | Cost sources |
| --- | --- | --- | --- | --- |
| *France* |  |  |  |  |
| Basson *et al.*, 2018, Diabetes Ther (doi: 10.1007/s13300-017-0321-0) | Compare long-term cost-effectiveness of dulaglutide versus once-weekly exenatide (type 2 diabetes) | SHI; direct medical costs  *Annual treatment costs*  2014 (2016 for drug costs) | Pharmacy, consumable, including needle and SMBG, costs | Assurance Maladie and national tariffs |
| Charbonnel *et al.*, 2018, Pharmacoecon Open (doi: 10.1007/s41669-017-0050-3) | Estimate direct healthcare costs of type 2 diabetes (type 2 diabetes) | SHI; reimbursed direct medical costs  *Average annual per capita costs*  2013 | Pharmacy, physician consultations/visits, nursing care, dental care, transportation, hospitalization costs | Hospital costs: DRG (ENC)  Non-hospital costs: EGB |
| Chevalier *et al.*, 2014, Arch Cadiovasc Dis (doi: 10.1016/j.acvd.2014.04.009) | Assess cost-effectiveness of treatment strategies for atrial fibrillation (atrial fibrillation) | SHI; direct medical costs  *Average quarterly or annual per-capita costs*  2011 | Home visit by nurse | Assurance Maladie |
| Detournay *et al.,* 2015, Méd Malad Métab (doi: 10.1016/S1957-2557(15)30065-1) | Assess costs of antidiabetic insulin therapy (type 2 diabetes) | SHI; direct medical costs  *Average annual per capita costs*  2013 | Pharmacy, dental care, transportation, hospitalization costs | EGB |
| François *et al.*, 2016, BMC Health Serv Res (doi: 10.1186/s12913-016-1620-2) | Assess economic burden of UTI in GP practices (UTI) | SHI; direct medical and indirect costs  *Per visit costs and hourly wages*  2012 (2010 for wage data) | Physician visit cost; hourly wages | Visit costs: EGB  Wages: INSEE |
| Guelfucci *et al.,* 2013, BMC Endocr Disord (doi: 10.1186/1472-6823-13-15) | Estimate annual diabetes-related expenditures on ambulatory care, by antidiabetic therapy (type 2 diabetes) | SHI; reimbursed direct medical costs  *Average annual per capita costs*  2005–2010 | Pharmacy, physician consultations/visits, nursing care, EMS costs | EGB |
| Hanaire *et al.,* 2016, Santé Publique (doi: 10.3917/spub.166.0781) | Estimate healthcare costs before and after initiation of insulin therapy (type 2 diabetes) | SHI; reimbursed direct medical costs  *Average annual per capita costs*  Cost year not reported | Pharmacy, dental care, EMS, hospitalization costs | EGB |
| Rapp *et al.*, 2018, Value Health (doi: 10.1016/j.jval.2017.09.019) | Evaluate resource use and societal costs of Alzheimer’s (dementia) | Society; direct medical and indirect costs  *Cost per visit*  2010 | Pharmacy, inpatient, outpatient, physician, nurse, transportation costs | ATIH, Assurance Maladie |
| Schuetz *et al.,* 2013, PLoS One (doi: 10.1371/journal.pone.0066454) | Assess cost-effectiveness of vascular health checks (eligible for vascular health check, aged 40–75 years) | “Governmental payer perspective” (SHI for France); direct medical costs  *Average cost per visit*  2011 | Outpatient visit | Not reported |
| *Germany* |  |  |  |  |
| Amorosi *et al.,* 2014, Europace (doi: 10.1093/europace/euu038) | Estimate budget impact of left atrial appendage closure (atrial fibrillation) | “German payer perspective”; direct medical costs  *Cost per visit*  2013 | Physician costs | GOÄ |
| Bock *et al.,* 2015, Gesundheitswesen (doi: 10.1055/s-0034-1374621) | Calculate unit costs for healthcare use (any) | Societal perspective  *Value per contact (physician), day (hospital, ICU, formal care)*  2011 | Physician, including primary and specialist care, hospital, including ICU, formal care costs | Top-down costing using SHI and hospital expenditure data |
| Freund *et al.,* 2016, Ann Intern Med (doi: 10.7326/M14-2403) | Evaluate protocol-based care management delivered by medical assistants in patients at high-risk of future hospitalization | Clinical staff wages  *Standard wages*  Not reported | Time spent by medical assistants (nurses) and physicians to develop patient-specific care plans | Wages (source not reported) |
| Grupp *et al.,* 2016, Health Policy (doi: 10.1016/j.healthpol.2015.11.005) | Estimate reference value for healthcare utilization and costs (medical and non-medical) (any) | Societal perspective, including direct and indirect costs  *Average per capita costs for 6 months*  2014 | Outpatient care, including GP and specialist care, (mobile) nursing service, hospitalization, sick leave costs | Medical costs: Bock et al., 2015  Non-medical costs: gross wages from Destatis  (resource use based on survey) |
| Icks *et al.,* 2013, Diabet Med (doi: 10.1111/dme.12263) | Estimate patients’ time costs attributable to diabetes care (any, patients with diabetes as subgroup) | Patient (time)  *Mean patient per year costs*  2011 | Lost productivity | Net wages  (resource use based on survey) |
| Jacob *et al.,* 2017, J Diabetes Sci Technol (doi: 10.1177/1932296816658746) | Analyze prescription patterns and costs of antidiabetic drug treatment (type 2 diabetes) | Costs of antihyperglycemic drugs in GP and diabetologist practice  *Average annual per capita costs*  2015 | Pharmacy | IMS Health Disease Analyzer database |
| Jacobs *et al.,* 2017, Diabet Med (doi: 10.1111/dme.13336) | Estimate direct diabetes-related costs (type 2 diabetes) | SHI; direct costs  *Average annual per capita costs*  2009–2010 | Pharmacy, physician, dental care, hospitalization, sick leave costs | Routine data from random sample of SHI-insured population |
| Köster *et al.,* 2014, Exp Clin Endocrinol Diabetes (doi: 10.1055/s-0034-1375675) | Estimate direct diabetes-related healthcare costs (diabetes) | SHI; direct costs  *Average annual per capita costs*  2010 | Pharmacy, hospitalization (physician cost as “physicians’ outpatient services”) | Routine data from SHI-insured patients in Hesse |
| Müller *et al.,* 2015, Diabet Med (doi: 10.1111/dme.12747) | Analyze diabetes-related healthcare utilization and costs (type 2 diabetes) | SHI; direct costs  *Average annual per capita costs*  2010 | Pharmacy, hospitalization costs | Routine data from SHI-insured patients |
| Neubauer *et al.,* 2018, Klin Monbl Augenheilkd (doi: 10.1055/s-0043-125076) | Model costs of intravitreal therapies for diabetic macular edema (macular edema) | SHI; direct costs  *Costs per injection or consultation*  2017 | Pharmacy, physician consultation costs | Pharmacy: Lauer-Taxe  Consultation: EBM |
| Schnell *et al.,* 2014, J Diabetes Sci Technol (doi: 10.1177/1932296813516206) | Model impact of improved accuracy of blood glucose meters | “Market prices”  *Average annual per capita costs*  Cost year not reported | SMBG costs | “Market prices” |
| Schuetz *et al.,* 2013, PLoS One (doi: 10.1371/journal.pone.0066454) | Assess cost-effectiveness of vascular health checks (eligible for vascular health check, aged 40–75 years) | “Governmental payer perspective” (SSN for Italy); direct medical costs  *Average cost per visit*  2011 | Outpatient visit costs | Not reported |
| Ulrich *et al.,* 2016, BMJ Open (doi: 10.1136/bmjopen-2016-012527) | Assess impact of type 2 diabetes on direct and indirect costs (type 2 diabetes) | Societal; direct and indirect costs  *Per visit and per day costs*  2011 | Pharmacy, outpatient care, including both primary and specialist care, hospitalization, sick leave costs | Pharmacy: WIdO  Outpatient and hospital costs: Bock et al. 2015  Sick leave: average labor costs as from Federal Statistical Office |
| Wilke *et al.,* 2016, Nephron (doi: 10.1159/000444420) | Assess costs of treatment for UTI in patients with diabetes (type 2 diabetes) | SHI; direct costs  *Costs per procedure (outpatient visits) and DRG (hospitalization)*  2010–2012 | Outpatient visits, including GP and specialists, hospitalization costs | Outpatient visits: EBM  Hospitalization: G-DRG |
| Wolff *et al.,* 2015, PLoS One (doi: 10.1371/journal.pone.0121910) | Analyze distribution of physicians’ time in eye hospital and costs (any) | Hospital; staff labor costs  *Average costs per patient-day in unit/ward*  2014 | Hospital ward, outpatient unit costs | Labor costs from hospital |
| *Italy* |  |  |  |  |
| Afonso *et al.,* 2017, J Med Econ (doi: 10.1080/13696998.2017.1304395) | Assess cost-effectiveness of lixisenatide versus insulin intensification (type 2 diabetes) | SSN; direct medical costs  *Costs per dose, lancet, test strip*  2016 | Pharmacy, consumables, SMBG costs | Pharmacy: Gazette Ufficiale and AIFA  Lancets: Lombardy  SMBG: SSR Veneto |
| Bruno *et al.,* 2016, Nutr Metab Cardiovasc Dis (doi: 10.1016/j.numecd.2016.09.002) | Assess incidence of type 1 diabetes and associated costs (type 1 diabetes) | SSN (various LHUs); direct medical costs  *Average annual per capita costs*  2012 | Pharmacy, consumables, outpatient visits, hospitalization costs | Pharmacy: “public prices reimbursed” by SSN  Outpatient visits, hospitalization: “regional tariffs”  No exact sources provided |
| Calò *et al.*, 2013, J Interv Card Electrophysiol (doi: 10.1007/s10840-013-9783-9) | Assess follow-up costs of remote monitoring of implanted defibrillators (implantable defibrillators) | Hospital and social costs of patients; direct medical costs  *Personnel cost per minute*  2012 | Nurse cost | Collective labor agreements, based on Istat data |
| Degli Esposti *et al.,* 2013, Clinicoecon Outcomes Res (doi: 10.2147/CEOR.S41846) | Assess association of glycemic control and healthcare costs (type 2 diabetes) | SSN (Tuscany and Emilia-Romagna); direct medical costs  *Average per costs over 2 years*  Cost year not reported | Pharmacy, hospitalization costs | Pharmacy: SSN purchase price  Hospitalization: DRGs from Emilia-Romagna and Tuscany |
| Degli Esposti *et al.,* 2014, Clinicoecon Outcomes Res (doi: 10.2147/CEOR.S63666) | Compare clinical cost outcomes by antidiabetic medication (uncontrolled type 2 diabetes) | SSN (Tuscany and Emilia-Romagna); direct medical costs  *Average annual per capita costs*  Cost year not reported | Pharmacy, outpatient care, hospitalization costs | Pharmacy: SSN purchase price  Outpatient services: “tariffs applied by the regions”  Hospitalizations: DRG (no exact sources) |
| Demurtas *et al.,* 2017, Prim Care Diabetes (doi: 10.1016/j.pcd.2017.06.001) | Assess direct diabetes-related healthcare costs (diabetes) | SSN (Veneto); direct medical costs  *Average annual per capita costs*  2012 | Pharmacy, outpatient care, hospitalization costs | “Inpatient and outpatients [sic] fees and drugs [sic] costs” |
| Germini *et al.*, 2018, Eur J Intern Med (doi: 10.1016/j.ejim.2018.01.010) | Assess costs of COPD exacerbations in the ED | SSN  *Costs per transport or visit*  Cost year not reported | EMS, ED costs | Ministry of Health |
| Giorda *et al.,* 2017, Nutr Metab Cardiovasc Dis (doi: 10.1016/j.numecd.2016.10.005) | Assess costs of hypoglycemia (diabetes) | SSN (various regions, including Lazio), direct and indirect costs  *Average costs per visit (physician, ED), hour (EMS), stay (hospitalization), work day (lost productivity)*  2016 | Physician home visit, EMS, ED visit, treatment, hospitalization, lost productivity costs | Home visit: GP Council tariffs  EMS: Sanita24 (source not reported)  ED: Mattoni SSN  Hospitalization: 2016 Ministry of Health report  Lost productivity: work day (no exact source) |
| Ippolito *et al.,* 2016, J Healthc Manag (PMID: 28319962) | Establish tariffs for home healthcare services using TDABC (home and palliative care) | SSN (Campania); physicians’ contracts  *Average cost per minute of work*  2016 | Nurse, dietician, psychologist costs | Work contracts  (time estimates based on profiling of home care) |
| Marchesini *et al.,* 2014, Nutr Metab Cardiovasc Dis (doi: 10.1016/j.numecd.2013.09.012) | Compare diabetes costs between Italians and migrants to Italy (diabetes) | SSN; direct medical costs  *Cost per patient-year*  2010 | Pharmacy, hospitalization cost | Pharmacy: not specified  Hospitalization: “refund paid to hospitals” (no exact source) |
| Pagano *et al.,* 2016, Nutr Metab Cardiovasc Dis (doi: 10.1016/j.numecd.2016.05.002) | Assess impact of diabetes on healthcare costs (diabetes) | SSN (various regions); direct medical costs  *Average annual per capita costs*  2012 | Pharmacy, hospitalization cost | Pharmacy: “drug prices […] reimbursed” by SSN  Hospitalization: regional tariffs |
| Parekh *et al.,* 2018, Diabetes Ther (doi: 10.1007/s13300-018-0418-0) | Assess budget impact of insulin-related hypo-glycemia (insulin-treated diabetes) | SSN (various regions); direct medical costs  *Costs per visit or call*  2017 | Specialist outpatient care | National tariffs |
| Pirolo *et al.,* 2016, Global Reg Health Technol Assess (doi: 10.5301/GRHTA.5000209) | Assess healthcare costs of diabetes (diabetes) | SSN (Veneto); direct medical costs  *Average annual per capita costs*  2013 | Pharmacy, specialist outpatient care, emergency care, hospitalization costs | Pharmacy: costs to LHU  Outpatient and emergency care: regional tariffs  Hospitalization: regional DRGs |
| Ravasio *et al.,* 2016, Global Reg Health Technol Assess (doi: 10.5301/grhta.5000229) | Assess treatment associated with canagliflozin, glimepiride and sitagliptin (type 2 diabetes) | SSN; direct medical costs  *Average annual per capita costs*  2014 | Pharmacy, consumables (SMBG) costs | Pharmacy: AIFA  Consumables: regional data and national average |
| Schuetz *et al.,* 2013, PLoS One (doi: 10.1371/journal.pone.0066454) | Assess cost-effectiveness of vascular health checks (eligible for vascular health check, aged 40–75 years) | “Governmental payer perspective”  *Average cost per visit*  2011 | Outpatient visit costs | Not reported |
| Veronese *et al.,* 2016, Nutr Metab Cardiovasc Dis (doi: 10.1016/j.numecd.2016.01.007) | Assess costs associated with severe hypo-glycemia (presenting at ED with severe hypoglycemia) | SSN (Emilia-Romagna); direct medical costs  *Average costs per call or visit*  2012 | EMS, ED visit, ICU, (overall) hospitalization costs | Emergency care: regional tariffs  Hospitalization: DRG |

AIFA, Agenzia Italiana Del Farmaco; ATIH, Agence Technique de l’Information sur l’Hospitalisation; ASST, Azienda Socio Sanitaria Territoriale; CoDiM, Cost of Diabetes Mellitus; COPD, Chronic Obstructive Pulmonary Disease; Destatis, German Federal Statistical Office; DRG, Diagnosis-Related Group; EGB, Échantillon Généraliste des Bénéficiaires; EBM, Einheitlicher Bewertungsmassstab; ED, Emergency Department; ENC, Échelle/Étude Nationale des Coûts; EMS, Emergency Medical Services; G-DRG, German Diagnosis-Related Group; GOÄ, Gebührenordnung für Ärzte; GP, General Practitioner; ICU, Intensive Care Unit; INSEE, Institut National de la Statistique et des Études Économicques; Istat, Istituto Nazionale di Statistica; LHU, Local Health Authority; SHI, Statutory Healthcare Insurance (France); SMBG, Self-Monitoring of Blood Glucose; SSN, Servizio Sanitario Nazionale; SSR, Servizio Sanitario Regionale; T2D, Type 2 Diabetes; TDABC, Time-Driven Activity-Based Costing; UTI, Urinary Tract Infection; WIdO, Wissenschaftliches Institut der Allgemeinen Ortskrankenkassen.

**Online Resource 6: Suggested cost dataset for France**

| Resource use item | Unit costs | | |
| --- | --- | --- | --- |
|  | Cost value | Source | Comment |
| *Primary and specialist outpatient care* | | | |
| Primary care physician/GP | EUR 25.00 per office visit (2019 values)  EUR 25.00 per home visit (2019 values)  EUR 25.00 per phone call (teleconsultation) (2019 values) | Ameli: [*Tarifs conventionnels des médecins généralistes en France métropolitaine*](https://www.ameli.fr/medecin/exercice-liberal/remuneration/tarifs-generalistes/tarifs-metropole): codes G, VG and TCG (for home visits, mileage and daytime allowances can be accounted for if sufficient data available) | Fee related to visit/consultation but not specific treatment  Also applicable to dieticians (usually general practitioners with qualifications in nutrition)  Diabetes education included in hospitalization costs (assumed EUR 12.6, based on four medical acts, for nurses qualified to deliver home visits) |
| Cardiologist | EUR 47.73 per office visit (2019 values) | Ameli: [*Tarifs conventionnels des médecins specialists en France métropolitaine*](https://www.ameli.fr/medecin/exercice-liberal/remuneration/tarifs-specialistes/metropole): code CSC | Fee related to visit/consultation but not specific treatment |
| Dentist | EUR 23.00 per office visit (2018 values) | Ameli: [*Soins et prothèses dentaires: vos remboursements*](https://www.ameli.fr/assure/remboursements/rembourse/soins-protheses-dentaires/soins-protheses-dentaires), visit to dental surgeon | Fee related to visit/consultation but not specific treatment |
| Dermatologist, diabetologist, nephrologist, ophthalmologist, podiatrist | EUR 25.00 per office visit (2019 values) | Ameli: [*Tarifs conventionnels des médecins specialists en France métropolitaine*](https://www.ameli.fr/medecin/exercice-liberal/remuneration/tarifs/tarifs-specialistes): code GS | Fee related to visit/consultation but not specific treatment |
| Neurologist/(neuro-) psychiatrists | EUR 39.00 per office visit (2019 values) | Ameli: [*Tarifs conventionnels des médecins specialists en France métropolitaine*](https://www.ameli.fr/medecin/exercice-liberal/remuneration/tarifs/tarifs-specialistes): code CNPSY | Fee related to visit/consultation but not specific treatment  Psychotherapy by psychotherapists in private practice not reimbursed by Ameli |
| Nurse (home visit) | EUR 8.80 per home visit (2018 values) | Ameli: [*Infirmier (exercice liberal)/facturation et remuneration/tarifs conventionnels: Les tarifs applicables en métropole, dans les départements d'outre-mer et à Mayotte*](https://www.ameli.fr/infirmier/exercice-liberal/facturation-remuneration/tarifs-conventionnels/tarifs) | 30 minutes assumed sufficient to cover measurement of glycemia and blood pressure, administration of one injection (assumed 2 x AMI costs) and travel costs (minimum EUR 2.50) based on expert opinion |
| *Hospital and inpatient care* |  |  |  |
| Daytime hospitalization | EUR 529.05 (2016 values) | [RTC](https://www.scansante.fr/applications/cout-dunites-doeuvre): *93415 – Unité d’Hospitalisation de Courte Durée (UHCD), Total des charges nettes majorées des SA auxiliaires* across all categories and sizes for activity type *MCO orienté médecine* and section type *Sections de médecine, chirurgie, obstétrique (MCO)* | If detailed procedural data are available, this estimate may be replaced with GHS costs |
| Non-intensive care, overnight stay | EUR 386.04 per day (2016 values) | [RTC](https://www.scansante.fr/applications/cout-dunites-doeuvre): *93413162 – Diabétologie, Total des charges nettes majorées des SA auxiliaires* across all categories and sizes for activity type *Toutes* (*all*) and section type *Sections de médecine, chirurgie, obstétrique (MCO)* | If detailed procedural data are available, this estimate may be replaced with GHS costs  This estimate is diabetes-specific but can also be obtained from RTC for other clinical fields |
| Intensive care, overnight stay | EUR 1,371.67 per day (2016 values) | [RTC](https://www.scansante.fr/applications/cout-dunites-doeuvre): *9341421 – Soins Intensifs Médicaux Total des charges nettes majorées des SA auxiliaires* across all categories and sizes for activity type *Toutes* (*all*) and section type *Sections de médecine, chirurgie, obstétrique (MCO)* | If detailed procedural data are available, this estimate may be replaced with GHS costs |
| *Emergency medical care* | | | |
| Emergency department | EUR 143.51 (2016 values) | [RTC](https://www.scansante.fr/applications/cout-dunites-doeuvre): *932112 – Accueil et Traitements des Urgences de Médecine, Total des charges nettes majorées des SA auxiliaires* across all categories and sizes for activity type *Toutes* (*all*) and section type *Sections de médecine, chirurgie, obstétrique (MCO)* | If detailed procedural data are available, this estimate may be replaced with GHS costs |
| EMS transport | EUR 533.49 per 30 minutes (2016 values) | [RTC](https://www.scansante.fr/applications/cout-dunites-doeuvre): *Q021 – SMUR Terrestre, Total des charges nettes majorées des SA auxiliaires* across all categories and sizes for activity type *Toutes* (*all*) and section type *Sections de médecine, chirurgie, obstétrique (MCO)* |  |
| Planned patient transport | EUR 57.37 per transport (2013 values) | Ameli: [*Ambulance: Les tarifs conventionnels*, *prise en charge*](https://www.ameli.fr/transporteur-sanitaire/exercice-professionnel/facturation/tarifs/ambulances-tarifs-conventionnels) | Mileage allowances for rural and urban settings also available |
| *Pharmacy and consumables* | | | |
| Pharmacy | Various | To be sourced according to specific study needs from publicly available sources such as [Thesorimed](https://theso.prod-un.thesorimed.org/monographie) |  |
| Consumables | Various | To be sourced according to specific study needs from publicly available sources such as [LPP](https://www.ameli.fr/etablissement-de-sante/exercice-professionnel/nomenclatures-codage/lpp) |  |
| *Intangible resource use* | | | |
| Gross hourly wage | EUR 25.19 per hour (2018 values) | OECD data for average annual wages divided by the average number of hours worked per year, which, for France in 2018, would be EUR 38,289 divided by 1,520h equaling EUR 25.19/h |  |

Reimbursement of different items not included. Costs were correct at the time of writing (February 2019) but are subject to change so may need to be updated, based on the codes provided, for further use.
Ameli, Assurance Maladie; GHS, Groupes Homogènes de Séjours; GP, General Practitioner; EMS, Emergency Medical Services; LPP, Liste des Produits et Prestations; OECD, Organization for Economic Co-operation and Development; RTC, Référentiel de Coût des Unités d'Oeuvres; SMIC, Salaire Minimum Interprofessionnel de Croissance; SMUR, Structure Mobile d'Urgence et de Reanimation.

**Online Resource 7: Suggested cost dataset for Germany**

| Resource use item | Unit costs | | |
| --- | --- | --- | --- |
|  | Cost value | Source | Comment |
| *Primary and specialist outpatient care* | | | |
| Primary care physician/general practitioner | EUR 64.41 per case (2018 values) | [KBV Honorarbericht](https://www.kbv.de/media/sp/Honorarbericht_Tabellen.xlsx) (salary report) Q1/2018 (published February 2019), category general medicine (“Allgemeinmediziner/hausärztliche Internisten”) | Costing could also be based on Bock *et al.*, 2015, Gesundheitswesen (EUR 20.06 per contact in 2011 values) or on the [EBM](https://www.kbv.de/html/online-ebm.php), e.g. sum of lump sum payments for items 03000 (basic fee for primary care visit by patients aged 55 to 75 years), 03040 (additional fee for provision of basic services and to item 03000), 03060 (additional fee for item 03040), 03061 (additional fee for item 03060), 03220 (additional fee for item 03000 for treatment of patient with at least one life-changing chronic disease) and 03221 (additional fee for item 03220)  Home visit: EUR 22.94 per visit (EBM item 01410) |
| Cardiologist | EUR 82.61 per case (2018 values) | [KBV Honorarbericht](https://www.kbv.de/media/sp/Honorarbericht_Tabellen.xlsx) (salary report) Q1/2018 (published February 2019), category cardiology (“Innere Medizin, SP, Kardiologie”) | Costing could also be based on Bock *et al.*, 2015, Gesundheitswesen (EUR 65.44 per contact in 2011 values) or the [EBM](https://www.kbv.de/html/online-ebm.php), e.g. sum of lump sum payments for items 13542 (basic fee for cardiologic visit by patients aged 60 years and older [13541 for patients aged 6–59 years]), 13543 (additional fee for provision of basic services) and 13544 (additional fee for item 13543) |
| Dentist | EUR 38.81 per case (2018 values) | GOZ, based on sum of lump sum payments for items 0010 (thorough examination to determine diseases of teeth, mount and jaw and to collect and record periodontal findings) and 0030 (preparation of written treatment schedule and charges based on findings and evaluation of any models), assumed markup of 2.3 | Costing could also be based on Bock *et al.*, 2015, Gesundheitswesen (EUR 55.87 per contact in 2011 values) |
| Dermatologist | EUR 38.51 per case (2018 values) | [KBV Honorarbericht](https://www.kbv.de/media/sp/Honorarbericht_Tabellen.xlsx) (salary report) Q1/2018 (published February 2019), category dermatology (“Dermatologie”) | Costing could also be based on Bock *et al.*, 2015, Gesundheitswesen (EUR 18.89 per contact in 2011 values) or the EBM, e.g. sum of lump sum payments for items 10212 (basic fee for dermatologic visit by patients aged 60 years and older [10211 for patients aged 6–59 years]), 10220 (additional fee for provision of basic services) and 10222 (additional fee for item 10220) |
| Diabetologist | EUR 83.90 per case (2018 values) | [KBV Honorarbericht](https://www.kbv.de/media/sp/Honorarbericht_Tabellen.xlsx) (salary report) Q1/2018 (published February 2019), category endocrinology (“Innere Medizin, SP Endokrinologie”) | Costing could also be based on Bock *et al.*, 2015, Gesundheitswesen (EUR 65.44 per contact in 2011 values) or the [EBM](https://www.kbv.de/html/online-ebm.php), e.g. sum of lump sum payments for items 13342 (basic fee for dermatologic visit by patients aged 60 years or older [13341 for patients aged 6–59 years]), 13344 (additional fee for provision of basic services) and 13346 (additional fee for item 13594) |
| Dietician | EUR 33 per visit (2018 values) | Techniker Krankenkasse (sickness fund) *Welche Kosten übernimmt die TK für eine Ernährungsberatung?*, (*Which costs are covered by TK for a nutrition coaching*?) maximum reimbursed average cost per visit over five visits, based on maximum reimbursement of EUR 45 for first visit (1 hour) and EUR 30 for second to fifth visit (0.5 hours each), assuming 5 visits | If dietary advice provided by physician, this would be covered by basic fees in the EBM ("Verzeichnis der nicht gesondert berechnungsfähigen Leistungen") |
| Nephrologist | EUR 783.35 per case (2018 values) | [KBV Honorarbericht](https://www.kbv.de/media/sp/Honorarbericht_Tabellen.xlsx) (salary report) Q1/2018 (published February 2019), category nephrology (“Innere Medizin, SP Nephrologie”) | Costing could also be based on Bock *et al.*, 2015, Gesundheitswesen (EUR 65.44 per contact in 2011 values) or the [EBM](https://www.kbv.de/html/online-ebm.php), e.g. sum of lump sum payments for items 13592 (basic fee for nephrologic visit by patients aged 60 years or older [13591 for patients aged 6–59 years]), 13594 (additional fee for provision of basic services) and 13596 (additional fee for item 13594) |
| Neurologist | EUR 64.37 per case (2018 values) | [KBV Honorarbericht](https://www.kbv.de/media/sp/Honorarbericht_Tabellen.xlsx) (salary report) Q1/2018 (published February 2019), category neurology (“Neurologie”) | Costing could also be based on Bock *et al.*, 2015, Gesundheitswesen (EUR 44.72 per contact in 2011 values) or the [EBM](https://www.kbv.de/html/online-ebm.php), e.g. sum of lump sum payments for items 16212 (basic fee for neurologic visit by patients aged 60 years or older [16211 for patients aged 6–59 years]), 13215 (additional fee for provision of basic services) and 16217 (additional fee for item 13594) |
| Ophthalmologist | EUR 55.65 per case (2018 values) | [KBV Honorarbericht](https://www.kbv.de/media/sp/Honorarbericht_Tabellen.xlsx) (salary report) Q1/2018 (published February 2019), category ophthalmology (“Augenheilkunde”) | Costing could also be based on Bock *et al.*, 2015, Gesundheitswesen (EUR 34.78 per contact in 2011 values) or the [EBM](https://www.kbv.de/html/online-ebm.php), e.g. sum of lump sum payments for items 06212 (basic fee for ophthalmologic visit by patients aged 60 years or older [06211 for patients aged 6–59 years]), 06220 (additional fee for provision of basic services) and 06222 (additional fee for item 13594) |
| Podiatrist | EUR 15.15 per visit per leg (2019 values) | [EBM](https://www.kbv.de/html/online-ebm.php) item 02311 for treatment of diabetic foot |  |
| Psychiatrist | EUR 84.40 per case (2018 values) | [KBV Honorarbericht](https://www.kbv.de/media/sp/Honorarbericht_Tabellen.xlsx) (salary report) Q1/2018 (published February 2019), category psychiatry (“Psychiatrie”) | Costing could also be based on Bock *et al.*, 2015, Gesundheitswesen (EUR 44.72 per contact in 2011 values) or the [EBM](https://www.kbv.de/html/online-ebm.php), e.g. the sum of lump sum payments for items 21212 (basic psychiatric fee for patients aged 60 years or older), 21215 (basic fee for psychiatrists/neurologists), 21218 (additional fee for item 21212), 21219 (additional fee for item 21218), 21225 (additional fee for item 21215), 21226 (additional fee for item 21225), 21227 (additional fee for item 21212) and 21228 (additional fee for item 21215) |
| Psychotherapist | EUR 423.14 per case (2018 values) | [KBV Honorarbericht](https://www.kbv.de/media/sp/Honorarbericht_Tabellen.xlsx) (salary report) Q1/2018 (published February 2019), category psychotherapy (“Summe Psychotherapeuten (ärztlich/psychologisch)”) | Costing could also be based on Bock *et al.*, 2015, Gesundheitswesen (EUR 78.08 per contact in 2011 values) or the [EBM](https://www.kbv.de/html/online-ebm.php), e.g. the sum of lump sum payments for items 22212 (basic psychotherapy fee for patients aged 60 years or older), 22216 (additional fee for item 22212), 22218 (additional fee for item 22216) and 22219 (additional fee to item 22212) |
| Diabetes education | EUR 7.79 per 10 minutes (2019 values) | Lump sum payment for [EBM](https://www.kbv.de/html/online-ebm.php) item 03355/13360/04590 (educating patient or caregiver on how to use real-time continuous glucose monitoring device) | Item can be charged a maximum of 10 times; no data available for diabetes educators in their own right (who are rare in German healthcare) |
| Nurse | EUR 15.55 per hour (2018 values) | Bundesärztekammer, 2017, Dt. Ärzteblatt: *[Gehaltstarifvertrag für Medizinische Fachangestellte/Arzthelferinnen](https://www.bundesaerztekammer.de/fileadmin/user_upload/downloads/pdf-Ordner/MFA/GTV_MFA_2017.pdf)* (Labor agreement covering medical assistants) (valid 2017-04-01 to 2019-03-31) | Average across monthly wages for nurses working full-time in physicians' offices across all levels of qualification and experience, divided by 167 hours of full-time work per month  Nurses generally do not perform home visits in Germany |
| *Hospital and inpatient care* | | | |
| Daytime hospitalization | EUR 374.33 per day (2011 values) | Bock *et al.*, 2015, Gesundheitswesen, *Bewertungssätze teilstationär in Euro je Tag, Allgemeine Krankenhäuser* |  |
| Non-intensive care, overnight stay | EUR 575.90 per day (2011 values) | Bock *et al.*, 2015, Gesundheitswesen, *Bewertungssätze vollstationär in Euro je Tag, Allgemeine Krankenhäuser* | Costing could also be based on sickness fund publications, e.g. using average daily costs (accounting for costs to hospitals while also accounting for hospital spending on teaching, research, etc.) of EUR 596 reported by AOK “[Fakten und Zahlen](https://aok-bv.de/imperia/md/aokbv/aok/zahlen/zuf_2017_web.pdf)” (“Facts and Numbers”) 2017 publication |
| Intensive care, overnight stay | EUR 1,337.72 per day (2011 values) | Bock *et al.*, 2015, Gesundheitswesen, *Bewertungs-sätze vollstationär in Euro je Tag, Intensivstation* |  |
| *Emergency medical care* | | | |
| Emergency department | EUR 125.71 per admission (2014 values) | DKGEV [*Gutachten zur ambulanten Notfallversorgung im Krankenhaus: Fallkostenkalkulation und Struktur-analyse*](https://www.dkgev.de/media/file/19401.2015-02-17_Gutachten_zur_ambulanten_Notfallversorgung_im_Krankenhaus_2015.pdf) (*Expertise on ambulatory emergency care in hospitals: calculating costs per case and structural analysis*) average costs per emergency admission | Based on cost data for 612,070 cases from 55 hospitals  Could also be costed using pre-hospital (“vorstationär”) [cases](https://www.bks.tu-berlin.de/fileadmin/fg241/Berliner_Krankenhaus-Seminar/SS_15/Vortrag_Schoepke_BKS_20.05.2015.pdf), yielding costs of EUR 226 per patient |
| EMS transport | EUR 609.44 per hour (2018 values) | Kreis Euskirchen [*Gebührensatzung Rettungsdienst 2018*](https://www.kreis-euskirchen.de/service/downloads/gefahrenvorsorge/5_322GebuehrSatzgRD.pdf) (*Fee schedule for Emergency Medical Services*), sum of ambulance and dispatch fee | Also available for other counties/regions; reimbursement by sickness funds depends on release/transport to hospital |
| Planned patient transport | EUR 258.37 per hour (2018 values) | Kreis Euskirchen [*Gebührensatzung Rettungsdienst 2018*](https://www.kreis-euskirchen.de/service/downloads/gefahrenvorsorge/5_322GebuehrSatzgRD.pdf) (*Fee schedule for Emergency Medical Services*), sum of transport vehicle and dispatch fee | Also available for other counties/regions |
| *Pharmacy and consumables* | | | |
| Pharmacy and consumables | Various | To be sourced according to specific study needs from sources such as [Lauer-Taxe](https://www.cgm.com/lauer-fischer/loesungen_lf/lauer_taxe_lf/lauer_taxe.de.jsp)^®^ or [Rote Liste](https://online.rote-liste.de/)^®^ | Databases are proprietary and require paid subscription |
| *Intangible resource use* | | | |
| Gross hourly wage | EUR 29.73 per hour (2018 values) | OECD data for average annual wages divided by the average number of hours worked per year, which, for Germany in 2018, would be EUR 40,522 divided by 1,363h equaling EUR 29.73/h |  |

Reimbursement of different items not included. Costs were correct at the time of writing (February 2019) but are subject to change so may need to be updated, based on the codes provided, for further use. Note that a case is not equal to a visit but instead covers a period of three months for reimbursement purposes.
AOK, Allgemeine Ortskrankenkasse; DKGEV, Deutsche Krankenhausgesellschaft (German Hospital Federation); EBM, Einheitlicher Bewertungsmassstab; GOZ, Gebührenordnung für Zahnärzte; KBV, Kassenärztliche Bundesvereinigung.

**Online Resource 8: Suggested cost dataset for Italy**

| Resource use item | Unit costs | | |
| --- | --- | --- | --- |
|  | Cost value | Source | Comment |
| *Primary and specialist outpatient care* | | | |
| Primary care physician/general practitioner/dentist | EUR 12.91 per office visit (1992 values)  EUR 25.82 per home visit (1992 values) | Ordine dei Medici Chirurghi e degli Odontoiatri Provincia di Latina (Association of Surgeons and Dentists in Latina Province): [*Tariffario minimo nazionale delle prestazioni medico-chirurgiche e odontoiatriche in Euro*](https://www.ordinemedici.bz.it/it/tariffario-minimo/?download=105) *(National minimum tariffs for services by surgeons and dentists)* | Also available from other Associations; consistent with data used recently by Giorda *et al.*, 2017, Nutr Metab Cardiovasc Dis  Of note, in Italy, GPs generally receive remuneration from the SSN based on the number of patients in their care and other fixed/flexible payments (Ferre *et al.*, 2014, Health Syst Transit)  Fee related to visit/consultation but not specific treatment; dentist visit not generally covered by SSN  No data available for cost of teleconsultation |
| Cardiologist | ER: EUR 23.00 for first visit (2019 values)  Bo: EUR 25.00 for first visit (2019 values)  Pu: EUR 20.66 for first visit (2019 values)  Um: EUR 33.00 for first visit (2019 values) | ER: [*Prestazioni di Assistenza Specialistica Ambulatoriale Emilia-Romagna*](https://salute.regione.emilia-romagna.it/documentazione/nomenclatore-tariffario-rer/nomenclatore_tariffario-2017/at_download/file/Nomenclatore%20tariffario%20regionale%20(in%20formato%20pdf)(al%2001-09-18).pdf), code 89.7A.3 (first visit to cardiologist)  Bo: [*Prestazioni di assistenza specialistica ambulatoriale e relative tariffe, elencate secondo le branche specialistich*e,](http://www.provinz.bz.it/gesundheit-leben/gesundheit/downloads/1_Prestazioni_di_specialistica_ambulatoriale_ex_DM_22.07.1996.pdf) based on code 89.7 (first visit to any specialist except neurologist, gynecologist, ophthalmologist)  Pu: [*Aggiornamento del Nomenclatore Tariffario e del Catalogo Unico Regionale*](http://www.sist.puglia.it/opencms/export/sites/sist/portale/files_download/NomenclatorePrestazioniSpecialisticheAmbulatoriali_-_20180301.xls), based on cade 89.7 (first visit)  Um: [*Tariffario Prestazioni specialistiche*](http://dati.umbria.it/dataset/977b04a2-5a14-47bc-ac2c-e9bb93f8deb3/resource/9243d66e-2245-4e52-869d-0f2304f52a46/download/exportprestazioni.csv), based on code 89.7A.3 (first visit to cardiologist) | Fee related to visit/consultation but not specific treatment |
| Dermatologist | ER: EUR 23.00 for first visit (2019 values)  Bo: EUR 25.00 for first visit (2019 values)  Pu: EUR 20.66 for first visit (2019 values)  Um: EUR 20.00 for visit (2019 values) | ER: [*Prestazioni di Assistenza Specialistica Ambulatoriale Emilia-Romagna*](https://salute.regione.emilia-romagna.it/documentazione/nomenclatore-tariffario-rer/nomenclatore_tariffario-2017/at_download/file/Nomenclatore%20tariffario%20regionale%20(in%20formato%20pdf)(al%2001-09-18).pdf), code 89.7A.7 (first visit to dermatologist)  Bo: [*Prestazioni di assistenza specialistica ambulatoriale e relative tariffe, elencate secondo le branche specialistich*e,](http://www.provinz.bz.it/gesundheit-leben/gesundheit/downloads/1_Prestazioni_di_specialistica_ambulatoriale_ex_DM_22.07.1996.pdf) based on code 89.7 (first visit to any specialist except neurologist, gynecologist, ophthalmologist)  Pu: [*Aggiornamento del Nomenclatore Tariffario e del Catalogo Unico Regionale*](http://www.sist.puglia.it/opencms/export/sites/sist/portale/files_download/NomenclatorePrestazioniSpecialisticheAmbulatoriali_-_20180301.xls), based on cade 89.7 (first visit)  Um: [*Tariffario Prestazioni specialistiche*](http://dati.umbria.it/dataset/977b04a2-5a14-47bc-ac2c-e9bb93f8deb3/resource/9243d66e-2245-4e52-869d-0f2304f52a46/download/exportprestazioni.csv), based on code 89.7 (general visit) | Fee related to visit/consultation but not specific treatment |
| Diabetologist | ER: EUR 23.00 for first visit (2019 values)  Bo: EUR 25.00 for first visit (2019 values)  Pu: EUR 20.66 for first visit (2019 values)  Um: EUR 20.00 for visit (2019 values) | ER: [*Prestazioni di Assistenza Specialistica Ambulatoriale Emilia-Romagna*](https://salute.regione.emilia-romagna.it/documentazione/nomenclatore-tariffario-rer/nomenclatore_tariffario-2017/at_download/file/Nomenclatore%20tariffario%20regionale%20(in%20formato%20pdf)(al%2001-09-18).pdf), code 897A80 (first visit to diabetologist)  Bo: [*Prestazioni di assistenza specialistica ambulatoriale e relative tariffe, elencate secondo le branche specialistich*e,](http://www.provinz.bz.it/gesundheit-leben/gesundheit/downloads/1_Prestazioni_di_specialistica_ambulatoriale_ex_DM_22.07.1996.pdf) based on code 89.7 (first visit to any specialist except neurologist, gynecologist, ophthalmologist)  Pu: [*Aggiornamento del Nomenclatore Tariffario e del Catalogo Unico Regionale*](http://www.sist.puglia.it/opencms/export/sites/sist/portale/files_download/NomenclatorePrestazioniSpecialisticheAmbulatoriali_-_20180301.xls), based on cade 89.7 (first visit)  Um: [*Tariffario Prestazioni specialistiche*](http://dati.umbria.it/dataset/977b04a2-5a14-47bc-ac2c-e9bb93f8deb3/resource/9243d66e-2245-4e52-869d-0f2304f52a46/download/exportprestazioni.csv), based on code 89.7 (general visit) | Fee related to visit/consultation but not specific treatment |
| Dietician | ER: *included in fee for diabetologist visit*  Bo: EUR 10.30 per visit (2019 values)  Um: EUR 8.50 per visit | ER: [*Prestazioni di Assistenza Specialistica Ambulatoriale Emilia-Romagna*](https://salute.regione.emilia-romagna.it/documentazione/nomenclatore-tariffario-rer/nomenclatore_tariffario-2017/at_download/file/Nomenclatore%20tariffario%20regionale%20(in%20formato%20pdf)(al%2001-09-18).pdf), code 897A80 (first visit to diabetologist)  Bo: [*Prestazioni di assistenza specialistica ambulatoriale e relative tariffe, elencate secondo le branche specialistich*e,](http://www.provinz.bz.it/gesundheit-leben/gesundheit/downloads/1_Prestazioni_di_specialistica_ambulatoriale_ex_DM_22.07.1996.pdf) based on code 93.01.5 (preparation of eating plan and control of progress)  Um: [*Tariffario Prestazioni specialistiche*](http://dati.umbria.it/dataset/977b04a2-5a14-47bc-ac2c-e9bb93f8deb3/resource/9243d66e-2245-4e52-869d-0f2304f52a46/download/exportprestazioni.csv), based on code 93.01.5 (nutritional counseling) | Fee related to visit/consultation but not specific treatment |
| Nephrologist | ER: EUR 23.00 for first visit (2019 values)  Bo: EUR 25.00 for first visit (2019 values)  Pu: EUR 20.66 for first visit (2019 values)  Um: EUR 20.00 for visit (2019 values) | ER: [*Prestazioni di Assistenza Specialistica Ambulatoriale Emilia-Romagna*](https://salute.regione.emilia-romagna.it/documentazione/nomenclatore-tariffario-rer/nomenclatore_tariffario-2017/at_download/file/Nomenclatore%20tariffario%20regionale%20(in%20formato%20pdf)(al%2001-09-18).pdf), code 89.7B.4 (first visit to nephrologist)  Bo: [*Prestazioni di assistenza specialistica ambulatoriale e relative tariffe, elencate secondo le branche specialistich*e,](http://www.provinz.bz.it/gesundheit-leben/gesundheit/downloads/1_Prestazioni_di_specialistica_ambulatoriale_ex_DM_22.07.1996.pdf) based on code 89.7 (first visit to any specialist except neurologist, gynecologist, ophthalmologist)  Pu: [*Aggiornamento del Nomenclatore Tariffario e del Catalogo Unico Regionale*](http://www.sist.puglia.it/opencms/export/sites/sist/portale/files_download/NomenclatorePrestazioniSpecialisticheAmbulatoriali_-_20180301.xls), based on cade 89.7 (first visit)  Um: [*Tariffario Prestazioni specialistiche*](http://dati.umbria.it/dataset/977b04a2-5a14-47bc-ac2c-e9bb93f8deb3/resource/9243d66e-2245-4e52-869d-0f2304f52a46/download/exportprestazioni.csv), based on code 89.7 (general visit) | Fee related to visit/consultation but not specific treatment |
| Neurologist | ER: EUR 23.00 for first visit (2019 values)  Bo: EUR 25.00 for first visit (2019 values)  Pu: EUR 20.66 for first visit (2019 values)  Um: EUR 20.00 for visit (2019 values) | ER: [*Prestazioni di Assistenza Specialistica Ambulatoriale Emilia-Romagna*](https://salute.regione.emilia-romagna.it/documentazione/nomenclatore-tariffario-rer/nomenclatore_tariffario-2017/at_download/file/Nomenclatore%20tariffario%20regionale%20(in%20formato%20pdf)(al%2001-09-18).pdf),, code 89.13 (first visit to neurologist)  Bo: [*Prestazioni di assistenza specialistica ambulatoriale e relative tariffe, elencate secondo le branche specialistich*e,](http://www.provinz.bz.it/gesundheit-leben/gesundheit/downloads/1_Prestazioni_di_specialistica_ambulatoriale_ex_DM_22.07.1996.pdf) based on code 89.13 (first visit to neurologist)  Pu: [*Aggiornamento del Nomenclatore Tariffario e del Catalogo Unico Regionale*](http://www.sist.puglia.it/opencms/export/sites/sist/portale/files_download/NomenclatorePrestazioniSpecialisticheAmbulatoriali_-_20180301.xls), based on cade 89.7 (first visit)  Um: [*Tariffario Prestazioni specialistiche*](http://dati.umbria.it/dataset/977b04a2-5a14-47bc-ac2c-e9bb93f8deb3/resource/9243d66e-2245-4e52-869d-0f2304f52a46/download/exportprestazioni.csv), based on code 89.13 (visit to neurologist) | Fee related to visit/consultation but not specific treatment |
| Ophthalmologist | ER: EUR 23.00 per visit (2019 values)  Bo: EUR 25.00 for first visit (2019 values)  Pu: EUR 20.66 for first visit (2019 values)  Um: EUR 20.00 for visit (2019 values) | ER: [*Prestazioni di Assistenza Specialistica Ambulatoriale Emilia-Romagna*](https://salute.regione.emilia-romagna.it/documentazione/nomenclatore-tariffario-rer/nomenclatore_tariffario-2017/at_download/file/Nomenclatore%20tariffario%20regionale%20(in%20formato%20pdf)(al%2001-09-18).pdf),, code 95.02 (full eye examination)  Bo: [*Prestazioni di assistenza specialistica ambulatoriale e relative tariffe, elencate secondo le branche specialistich*e,](http://www.provinz.bz.it/gesundheit-leben/gesundheit/downloads/1_Prestazioni_di_specialistica_ambulatoriale_ex_DM_22.07.1996.pdf) based on code 95.02 (full eye examination)  Pu: [*Aggiornamento del Nomenclatore Tariffario e del Catalogo Unico Regionale*](http://www.sist.puglia.it/opencms/export/sites/sist/portale/files_download/NomenclatorePrestazioniSpecialisticheAmbulatoriali_-_20180301.xls), based on cade 95.02 (full eye examination)  Um: [*Tariffario Prestazioni specialistiche*](http://dati.umbria.it/dataset/977b04a2-5a14-47bc-ac2c-e9bb93f8deb3/resource/9243d66e-2245-4e52-869d-0f2304f52a46/download/exportprestazioni.csv), based on code 95.02 (full eye examination) | Fee related to visit/consultation but not specific treatment |
| Podiatrist | Bo: EUR 25.00 for first visit (2019 values) | Bo: [*Prestazioni di assistenza specialistica ambulatoriale e relative tariffe, elencate secondo le branche specialistich*e,](http://www.provinz.bz.it/gesundheit-leben/gesundheit/downloads/1_Prestazioni_di_specialistica_ambulatoriale_ex_DM_22.07.1996.pdf) based on code 1300.02 (simple podologic treatment) |  |
| Psychiatrist | ER: EUR 23.00 per visit (2019 values)  Bo: EUR 25.00 for visit (2019 values)  Pu: EUR 19.37 for visit (2019 values)  Um: EUR 20.00 for visit (2019 values) | ER: [*Prestazioni di Assistenza Specialistica Ambulatoriale Emilia-Romagna*](https://salute.regione.emilia-romagna.it/documentazione/nomenclatore-tariffario-rer/nomenclatore_tariffario-2017/at_download/file/Nomenclatore%20tariffario%20regionale%20(in%20formato%20pdf)(al%2001-09-18).pdf),, code 94.19.1 (psychiatric session)  Bo: [*Prestazioni di assistenza specialistica ambulatoriale e relative tariffe, elencate secondo le branche specialistich*e,](http://www.provinz.bz.it/gesundheit-leben/gesundheit/downloads/1_Prestazioni_di_specialistica_ambulatoriale_ex_DM_22.07.1996.pdf) based on code 94.19.1 (psychiatric session)  Pu: [*Aggiornamento del Nomenclatore Tariffario e del Catalogo Unico Regionale*](http://www.sist.puglia.it/opencms/export/sites/sist/portale/files_download/NomenclatorePrestazioniSpecialisticheAmbulatoriali_-_20180301.xls), based on code 94.19.1 (psychiatric session)  Um: [*Tariffario Prestazioni specialistiche*](http://dati.umbria.it/dataset/977b04a2-5a14-47bc-ac2c-e9bb93f8deb3/resource/9243d66e-2245-4e52-869d-0f2304f52a46/download/exportprestazioni.csv), based on code 94.19.1 (psychiatric session) |  |
| Psychotherapist | ER: EUR 19.35 per visit (2019 values)  Bo: EUR 19.40 for visit (2019 values)  Pu: EUR 19.37 for visit (2019 values)  Um: EUR 21.30 for visit (2019 values) | ER: [*Prestazioni di Assistenza Specialistica Ambulatoriale Emilia-Romagna*](https://salute.regione.emilia-romagna.it/documentazione/nomenclatore-tariffario-rer/nomenclatore_tariffario-2017/at_download/file/Nomenclatore%20tariffario%20regionale%20(in%20formato%20pdf)(al%2001-09-18).pdf),, code 94.3 (psychotherapy session)  Bo: [*Prestazioni di assistenza specialistica ambulatoriale e relative tariffe, elencate secondo le branche specialistich*e,](http://www.provinz.bz.it/gesundheit-leben/gesundheit/downloads/1_Prestazioni_di_specialistica_ambulatoriale_ex_DM_22.07.1996.pdf) based on code 94.3 (psychotherapy session)  Pu: [*Aggiornamento del Nomenclatore Tariffario e del Catalogo Unico Regionale*](http://www.sist.puglia.it/opencms/export/sites/sist/portale/files_download/NomenclatorePrestazioniSpecialisticheAmbulatoriali_-_20180301.xls), based on code 94.3 (psychotherapy session)  Um: [*Tariffario Prestazioni specialistiche*](http://dati.umbria.it/dataset/977b04a2-5a14-47bc-ac2c-e9bb93f8deb3/resource/9243d66e-2245-4e52-869d-0f2304f52a46/download/exportprestazioni.csv), based on code 94.3 (psychotherapy session) |  |
| Diabetes education | ER: EUR 4.40 per visit (2019 values)  Bo: EUR 4.40 per visit (2019 values)  Pu: EUR 4.39 for visit (2019 values)  Um: EUR 4.80 for visit (2019 values) | ER: [*Prestazioni di Assistenza Specialistica Ambulatoriale Emilia-Romagna*](https://salute.regione.emilia-romagna.it/documentazione/nomenclatore-tariffario-rer/nomenclatore_tariffario-2017/at_download/file/Nomenclatore%20tariffario%20regionale%20(in%20formato%20pdf)(al%2001-09-18).pdf),, code 93.82.1 (diabetes, obesity education)  Bo: [*Prestazioni di assistenza specialistica ambulatoriale e relative tariffe, elencate secondo le branche specialistich*e,](http://www.provinz.bz.it/gesundheit-leben/gesundheit/downloads/1_Prestazioni_di_specialistica_ambulatoriale_ex_DM_22.07.1996.pdf) based on code 93.82.1 (diabetes education in individual session)  Pu: [*Aggiornamento del Nomenclatore Tariffario e del Catalogo Unico Regionale*](http://www.sist.puglia.it/opencms/export/sites/sist/portale/files_download/NomenclatorePrestazioniSpecialisticheAmbulatoriali_-_20180301.xls), based on code 93.82.1 (diabetes education)  Um: [*Tariffario Prestazioni specialistiche*](http://dati.umbria.it/dataset/977b04a2-5a14-47bc-ac2c-e9bb93f8deb3/resource/9243d66e-2245-4e52-869d-0f2304f52a46/download/exportprestazioni.csv), based on code 93.82.1 (diabetes education) |  |
| Nurse (home visit) | EUR 1.20 per visit (2019 values) | Bo: [*Prestazioni di assistenza specialistica ambulatoriale e relative tariffe, elencate secondo le branche specialistich*e,](http://www.provinz.bz.it/gesundheit-leben/gesundheit/downloads/1_Prestazioni_di_specialistica_ambulatoriale_ex_DM_22.07.1996.pdf) based on code 3183 (home control visit by nurse) | Fee related to visit/consultation but not specific treatment |
| *Hospital and inpatient care* |  |  |  |
| Daytime hospitalization | EUR 220.00 (2012 values) | Veronese *et al.*, 2016, Nutr Metab Cardiovasc Dis, cost for short-term observation, based on “regional cost book” for ER |  |
| Non-intensive care with overnight stay (per episode) | EUR 4,455 per admission (2012 values) | Veronese *et al.*, 2016, Nutr Metab Cardiovasc Dis, cost for non-intensive care stay in endocrinology department following severe hypoglycemia |  |
| Intensive care with overnight stay (per episode) | EUR 7,688 per admission (2012 values) | Veronese *et al.*, 2016, Nutr Metab Cardiovasc Dis, cost for critical care stay following severe hypoglycemia |  |
| *Emergency medical care* | | | |
| Emergency department | EUR 23.00 per visit (2012 values) | Veronese *et al.*, 2016, Nutr Metab Cardiovasc Dis, cost for ED visit |  |
| EMS transport | EUR 205.00 per call (2012 values) | Veronese *et al.*, 2016, Nutr Metab Cardiovasc Dis, cost for ambulance call |  |
| Planned patient transport | Lo: EUR 53.00 per return journey (2019 values)  La: EUR 40.00 per return journey (2019 values) | Lo: Croce Rossa Italiana Comitato di Gallarate: [*Rimborsi massimi da riconoscersi per i trasporti sanitari che non rivestono carattere di urgenza e emergenza resi direttamente a cittadini*](http://www.crigallarate.it/sites/default/files/documenti/Nuove%20Tariffe%20Servizi%20Secondari.pdf)*,* flat rate for car transport with one staff (“Auto a 1 soccorritore”)  La: Croce Rossa Italiana Comitato di Gallarate di Itri: [*Tariffario per trasporti socio-sanitari non urgenti*](http://www.cri-itri.it/wp-content/uploads/2016/09/Clicca-qui-per-scaricare-le-nostre-tariffe-2016.pdf), flat rate for local transport (“Trasporto infermo in ambito locale”) |  |
| *Pharmacy and consumables* | | | |
| Pharmacy | Various | To be sourced according to specific study needs from public sources such as [AIFA pharmacy database](https://farmaci.agenziafarmaco.gov.it/bancadatifarmaci/home) and the [Federfarma (pharmacy association) database](https://www.federfarma.it/Farmaci-e-farmacie/Cerca-un-farmaco.aspx) |  |
| Consumables | Various | To be sourced according to specific study needs from public *Costi unitari dispositivi medici*, e.g. for [institutions](http://www.asst-rhodense.it/SalviniWEB/AmministrazioneTrasparente/DisposizioniGenerali/DispositiviMedici.html) in Lo or institutions in [Sicily](https://www.ospedaliriunitipalermo.it/prezzi_dispositivi_medici.html) |  |
| *Intangible resource use* | | | |
| Gross hourly wage | EUR 17.18 per hour (2018 values) | OECD data for average annual wages divided by the average number of hours worked per year, which, for Italy in 2018, would be EUR 29,601 divided by 1,723h equaling EUR 17.18/h |  |

Reimbursement of different items not included. Costs were correct at the time of writing (February 2019) but are subject to change so may need to be updated, based on the codes provided, for further use. Tariffs and fees from the latest available source were assumed to apply in the present and therefore listed as 2019 values. Costs sourced from regions across Italy, including the northern (Bolzano), middle (Emilia-Romagna and Umbria) and southern (Apulia) part (data or other provinces are also available on the respective province websites, which usually use the same codes as those applied here).
AIFA, Agenzia Italiana del Farmaco; Bo, Bolzano; ER, Emilia-Romagna; GP, General Practitioner; DRG, Diagnosis-Related Group; LA, Lazio; Lo, Lombardy; OECD, Organization for Economic Co-operation and Development; Pu, Puglia (Apulia); SSN, Servizio Sanitario Nazionale; Um, Umbria.
